# Mass Spectrometry-Based Targeted Lipidomics and Supervised Machine Learning Algorithms in Detecting Disease, Cultivar, and Treatment Biomarkers in *Xylella fastidiosa* subsp. *pauca*-Infected Olive Trees

**DOI:** 10.3389/fpls.2022.833245

**Published:** 2022-04-22

**Authors:** Valeria Scala, Manuel Salustri, Stefania Loreti, Nicoletta Pucci, Andrea Cacciotti, Giuseppe Tatulli, Marco Scortichini, Massimo Reverberi

**Affiliations:** ^1^Council for Agricultural Research and Economics (CREA), Research Centre for Plant Protection and Certification, Rome, Italy; ^2^Department of Environmental Biology, Sapienza University, Rome, Italy; ^3^Council for Agricultural Research and Economics (CREA), Research Centre for Olive, Fruit and Citrus Crops, Rome, Italy

**Keywords:** *Xylella fastidiosa*, olive trees, lipids, machine learning algorithms, oxylipins, detection

## Abstract

In 2013, *Xylella fastidiosa (Xf)* was detected for the first time in Apulia and, subsequently, recognized as the causal agent of the olive quick decline syndrome (OQDS). To contain the disease, the olive germplasm was evaluated for resistance to *Xf*, identifying cultivars with different susceptibility to the pathogen. Regarding this, the resistant cultivar Leccino has generally a lower bacterial titer compared with the susceptible cultivar Ogliarola salentina. Among biomolecules, lipids could have a pivotal role in the interaction of *Xf* with its host. In the grapevine Pierce’s disease, fatty acid molecules, the diffusible signaling factors (DSFs), act as regulators of *Xf* lifestyle and are crucial for its virulence. Other lipid compounds derived from fatty acid oxidation, namely, oxylipins, can affect, *in vitro*, biofilm formation in *Xf* subsp. *pauca (Xfp)* strain De Donno, that is, the strain causing OQDS. In this study, we combined high-performance liquid chromatography-mass spectrometry-MS-based targeted lipidomics with supervised learning algorithms (random forest, support vector machine, and neural networks) to classify olive tree samples from Salento. The dataset included samples from either OQDS-positive or OQDS-negative olive trees belonging either to cultivar Ogliarola salentina or Leccino treated or not with the zinc-copper-citric acid biocomplex Dentamet^®^. We built classifiers using the relative differences in lipid species able to discriminate olive tree samples, namely, (1) infected and non-infected, (2) belonging to different cultivars, and (3) treated or untreated with Dentamet^®^. Lipid entities emerging as predictors of the thesis are free fatty acids (C16:1, C18:1, C18:2, C18:3); the LOX-derived oxylipins 9- and 13-HPOD/TrE; the DOX-derived oxylipin 10-HPOME; and diacylglyceride DAG36:4(18:1/18:3).

## Introduction

*Xylella fastidiosa* (*Xf*) was detected for the first time in Europe in the Salento area of Apulia, and, subsequently, it was recognized as the causal agent of a novel devastating disease, representing a serious threat to the local agriculture economy and biodiversity, that is, the olive quick decline syndrome (OQDS). This disease was characterized by severe branch desiccation and rapid death of olive trees. Initial field observations tentatively attributed this novel and rapid-spreading disease to a variety of biotic and abiotic causes (Martelli, 2016), before revealing the presence of the quarantine pathogen *Xf* subsp. *pauca (Xfp)* strain De Donno (Saponari et al., 2013). The most severely affected olives are the century-old trees of local cultivars (cvs) Cellina di Nardò and Ogliarola salentina, which are highly susceptible (Saponari et al., 2019), cv Leccino shows mild branch dieback despite being adjacent to or close to orchards with severe OQDS symptoms (Giampetruzzi et al., 2016; Saponari et al., 2019).

To unveil the molecular events involved in this interaction, a transcriptome profiling of the xylem tissues from twigs of *Xfp-*naturally infected and non-infected plants of both cultivars was conducted. *Xfp* is perceived and elicits a different transcriptome response in the two cultivars; currently, cv Leccino upregulates genes encoding receptor-like kinases and receptor-like proteins, involved in pathogen recognition, whereas cv Ogliarola salentina does not (Giampetruzzi et al., 2016). Most recently, *Xfp* artificial infection of the model plant *Nicotiana* spp. activates hydrolases/hydrolase inhibitors, serine proteases, metal transferases, and triggers cell death-like phenotypes in plants (Sertedakis et al., 2021). Alongside transcriptomics, metabolomic studies focused on OQDS are gaining momentum and find some secondary metabolites as infection biomarkers (Girelli et al., 2019; Nicolì et al., 2019; Vergine et al., 2020). These analyses fail to consider lipid classes as an important target to study this interaction. Lipids appear to be central in the interaction *Xf* displayed with its host. In the literature, it is reported that lipids, such as *cis*-2-monounsaturated fatty acids, named diffusible signaling factors (DSFs) act as regulators of *Xf* lifestyle in Pierce’s disease of the grapevine. Specifically, DSFs allow the switch from a planktonic stage, typical of endophytic lifestyle and useful to spread within xylem vessels, to a sessile stage, in which the pathogen can form a biofilm that eventually blocks the xylem sap flux. As stated above, this mechanism has been proved regarding the grapevine Pierce’s disease caused by *Xf* subsp. *fastidiosa* strain Temecula1 (Newman et al., 2004; Chatterjee et al., 2008; Lindow et al., 2014; Ionescu et al., 2016), and it is not unlikely that *Xfp* strain De Donno provokes OQDS symptoms in the same way (Scala et al., 2020). In *Pseudomonas aeruginosa*, other lipids, namely, the Oxylipins, act as hormones for controlling the switch among the different stages of the bacterial lifestyle, namely, dispersal and adherent biofilm phases (Martínez and Campos-Gómez, 2016; Martínez et al., 2019) and noteworthy, oxylipins, *in vitro*, affect biofilm formation in *Xfp* strain De Donno (Scala et al., 2020). In addition, 10 lipid entities are recognized as possible OQDS infection biomarkers in Ogliarola salentina. These molecules are identified through subsequent use of untargeted and targeted high-performance liquid chromatography (HPLC) coupled mass spectrometry, namely, they are oxylipins deriving from lipoxygenase (LOX) and dioxygenase (DOX) activity, free fatty acids, and diacylglycerols (Scala et al., 2020). LOX and DOX catalyze the polyunsaturated fatty acid (PUFA) dioxygenation. Oxygenation may occur at several positions along the carbon chain.

In this study, we combined high-performance liquid chromatography-mass spectrometry (HPLC-MS)-based targeted lipidomics with supervised learning algorithms, that is, random forest (RF), support vector machine (SVM), and neural networks (NNet), to assess in robust manner biomarkers for different conditions in the field. The analyzed dataset included olive tree samples from three different places in Apulia divided equally between cv Ogliarola salentina and cv Leccino. The samples are (1) asymptomatic trees, (2) symptomatic trees, and (3) symptomatic trees treated with Dentamet^®^. Dentamet^®^ is a citric acid-zinc-copper-based mixture, a promising biocomplex to control OQDS symptoms (Scortichini et al., 2018). We hypothesized that the relative differences in some lipid species, selected according to our previous studies, can be used to discriminate among olive tree samples (1) positive or negative to *Xf* infection, regardless of the cultivar; (2) belonging to different cultivars; and (c) treated or untreated with Dentamet^®^. The ability to discriminate diseased and healthy plants through lipid entities opens different scenarios, namely, employ these molecules as biochemical diagnostic markers, develop a non-destructive diagnosis tool (since oxylipins are precursors of volatile organic compounds), and apply these molecules in defense strategies.

## Materials and Methods

### Study Site and Sampling Procedures

Sampling was carried out in the Salento area, in July 2020, on 66 individuals of *Olea europaea* cv Ogliarola salentina (O) and cv Leccino (L), from three locations, namely, Nardò, Galatone (Lecce province), and Lizzano (Taranto province), in Apulia region, whose coordinates are 40°24′06.7″N 17°26′10.4″E, 40°11′19.2″N 18°01′18.3″E, 40°09′19.1″N 18°03′44.0″E; an equal number of samples for each cultivar were collected (Table 1). The design was completely randomized. Trees were, respectively, OQDS asymptomatic (OQDS-), showing OQDS symptoms (OQDS +) and OQDS + treated with Dentamet^®^. Trees were identified as symptomatic or asymptomatic following the criteria previously reported (Giampetruzzi et al., 2016). The samples consisted of 1- or 2-year-old twigs (*ca*. 0.5 cm in diameter) from which cuttings of 15–20 cm were prepared. Concerning symptomatic trees, cuttings come from “the portions close but unaffected by the withering and desiccation phenomena” (Giampetruzzi et al., 2016). Xylem tissue was then recovered, after removing the bark, and processed. The obtained 66 samples were separately lyophilized. Samples OQDS +, OQDS-, and OQDS + treated with Dentamet^®^ were molecularly assayed *via* real-time PCR (Harper et al., 2010, European Plant and Protection Organization, 2016) in three technical replicates to verify the presence of *Xf.*

**TABLE 1:** Summary of X, H, and X treated with Dentamet^®^ samples. X denotes samples in which *X. fastidiosa* is present, H denotes samples in which *X. fastidiosa* is absent, DX indicates olive trees treated with Dentamet^®^ in which *X. fastidiosa* is present.

Cultivar	X	DX	H
Leccino (L)	14	11	8
Ogliarola (O)	12	11	10
Total	26	22	18

### High-Performance Liquid Chromatography-Mass Spectrometry/MS

Xylem tissue (1.0 g) was recovered, and lipids extraction and analysis were performed as previously reported (Scala et al., 2018). Samples were assayed with the internal reference standards glyceryl tripalmitate d31 and 9-HODEd4. The analysis was carried out at a final concentration of 2 μM. The samples were analyzed (fragmentation analysis) by LC-MS/MS (Triple Quadrupole; 6420 Agilent Technologies, United States), adopting multiple reaction monitoring (MRM) methods following Scala et al. (2018). The mass spectrometry analyses were performed in technical duplicate. MRM data were processed using the Mass Hunter Quantitative software (B.07.00 version, Agilent Technologies, United States). Integrated peak areas were normalized upon Internal Standard Peak Area/max (Internal Standard Peak Area). Several comparisons were assessed among samples named *Xf* positive vs. *Xf* negative (X vs. H); cv Ogliarola salentina *Xf* positive vs. cv Ogliarola salentina *Xf* negative (OX vs. OH); cv Leccino *Xf* positive vs. cv Leccino *Xf* negative (LX vs. LH); cv Ogliarola salentina vs. cv Leccino (O vs. L); cv Ogliarola salentina *Xf* positive vs. cv Leccino *Xf* positive (OX vs. LX); cv Ogliarola salentina *Xf* negative vs. cv Leccino *Xf* negative (OH vs. LH); cv Ogliarola salentina *Xf* positive and cv Leccino *Xf* positive treated with Dentamet^®^ vs. cv Ogliarola salentina *Xf* positive and cv Leccino *Xf* positive untreated with Dentamet^®^ (DX vs. NDX).

### Bioinformatic Analysis of RNAseq Data

RNAseq analysis was performed on data published in Giampetruzzi et al. (2016) obtained from three *Xf*-infected Ogliarola salentina (OX), three *Xf-*infected Leccino (LX), two healthy Ogliarola salentina (OH), and two healthy Leccino (LH). The files available online^1^ were used for our targeted transcriptome analysis. Bioconductor’s DESeq2 R package 1.10.1 was used to evaluate the differential expression. The software uses the Benjamini and Hochberg procedure to obtain the *p*-values indicative of the false discovery rate. *P*-values less than 0.05 were used to identify differentially expressed genes. The differential gene expression was analyzed in X vs. H, OX vs. OH, LX vs. LH, O vs. L, OX vs. LX, and OH vs. LH.

### Statistics and Machine Learning

Univariate statistical analysis and machine learning methods were used to assess differences in lipid profiles for each comparison, aiming at identifying biomarkers for the different conditions (*Xf* positivity, cultivar, Dentamet^®^ treatment). Univariate statistical analysis consisted the calculation of fold change and *p*-values (Wilcoxon Mann-Whitney test) for each lipid compound between two conditions. Biomarker candidates were identified as most statistically significant compounds (*p*-value < 0.05) and most fold-changed compounds (fc ≥ 1.5 vs. fc ≤ 1/1.5). For machine learning analysis, normalized compound areas were scaled and centered. Biomarker candidates were identified through recursive feature elimination based on RF algorithm using five times 10-fold-cross-validation to validate results, and setting the search for the 10 best predictors. Predictors were used in constructing supervised learning models for each comparison. RF, SVM, and NNet were chosen. Each model was trained on a 60% subset of the whole dataset, and validation of the training set was performed through 10-fold-cross-validation repeated five times. Features’ importance was evaluated. Receiver-operating characteristic curves were computed, to compare models, to identify the best probability threshold, and above all to assess the goodness of fit of the model. The trained models were applied on test sets, corresponding to the 40% of datasets not used in training. Metrics were computed on models applied on test sets to confirm their performances. Analysis was performed through R (version 4.0.2), using packages “stats” (R Core Team, 2020) and “caret” (Kuhn, 2020). Dataset splitting, machine learning pipeline, and metrics computation are graphically explained in Supplementary Figures 1–3.

## Results

### Samples Classification

The whole dataset (66 specimens equally belonging to cv Ogliarola salentina, O, and cv Leccino, L) sampled in three localities of Apulia (Nardò, Lizzano, and Galatone) were clustered as OQDS +, that is, olive trees showing symptoms of OQDS (sampled in Nardò); OQDS-, asymptomatic olive trees (sampled in Lizzano) and olive trees showing symptoms of OQDS and treated with Dentamet^®^ (sampled in Galatone). We aimed to classify these samples according to the presence or absence of *Xf*. This was assessed by real-time PCR (Harper et al., 2010). Results (Table 1) indicated that in 26 OQDS + samples (14 L and 12 O) *Xf* was present (from here named LX or OX); in 22 samples treated with Dentamet^®^ (11 L and 11 O) *Xf* was present (from here named DX); in 18 OQDS- samples (8 L and 10 O) *Xf* was absent (from here named LH or OH).

### Lipidomic Analysis

#### *Xf*-positive vs. *Xf*-negative (X vs. H): Oxylipins, Diffusible Signaling Factor, and DAG as Hallmarks of Infection

**FIGURE 1:**
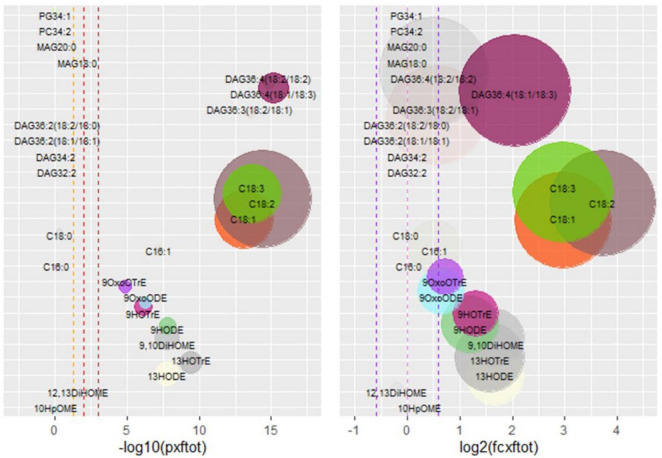
*Xf*-positive vs. *Xf*-negative (X vs. H) analysis of the lipid dataset. The left panel shows statistically significant compounds: on the x-axis, there is the −log10(*p*-value), on the y-axis compound names. The vertical dashed lines correspond to, respectively, −log10(0.05), −log10(0.01), and −log10(0.001). Compounds under the first threshold are represented as transparent, compounds above the threshold are in full colors. Circle dimensions are proportional to fold changes. On the right panel, x-axis there is the log2(fold-change), on the y-axis compound names. The right panel points out compounds with the biggest fold changes, represented in full colors, while compounds with a fold change ≥1.5 or ≤1.5 are transparent. Circle dimension is proportional to −log10(*p*-value). RF-based feature selection is used to obtain the top five predictors for machine learning analysis and, after five times 10-fold-cross-validation, the chosen features are indicated in Supplementary Figure 4 and Supplementary Table 2. These features are used to train three learning models based on different algorithms: RF, SVM, and NNet. The models are validated through 10-fold-cross-validation five times, and they all are performing well, with AUCs > 0.9: RF is the best model (Supplementary Figures 5A–C). The importance of the selected features (Supplementary Table 2) in X vs. H classification task is confirmed by applying the trained models on the test set with a default threshold of 0.5. The metrics for RF are very good, while SVM and NNET are less performing even though significant (Supplementary Table 1). phosphatidylglycerol (PG), phosphatidylcholine (PC), monoacylglycerol (MAG), diacylglycerol (DAG), stearic acid (C18:0), oleic acid (C18:1), linoleic acid (C18:2), linolenic acid (C18:3), palmitic acid (C16:0), palmitoleic acid (C16:1), oxo-octadecatrienoic acid (OxoOTrE), oxo-octadecenoic acid (OxoODE), hydroperoxyoctatrienoic acid (HOTrE), hydroxyoctadecenoic acid (HODE), (DiHOME), hydroperoxyoctamonoenoic acid (HpOME). Notation for FAs and oxylipins (OM/D/TrE) is reported as indicating the carbon number (CN) and the number of double bond (DB) equivalents (e.g., C18:1 is oleic acid and HODE is hydroxyoctadecenoic).

The RF, SVM, and NNeT models chosen for the analysis of the lipid dataset are reported in Supplementary Table 1. Statistically significant compounds (Table 2 and Figure 1) are 9-LOX derived oxylipins [9-hydroxyoctadecenoic acid (9-HODE), 9-hydroperoxyoctatrienoic acid (9-HOTrE), 9-OxoODE, 9-OxoOTrE, 9,10-DiHOME], 13-LOX-derived oxylipins [13-hydroxyoctadecenoic acid (13-HODE), 13-hydroperoxyoctatrienoic acid (13-HOTrE)], free fatty acids (C16:1, C18:1, C18:2, C18:3), and diacylglycerols (DAG36:4 with two C18:2 or with C18:1/C18:3 moieties, DAG36:3 with C18:1/C18:2 moieties). The chosen features for machine learning are reported in Supplementary Figure 4 and Supplementary Table 2. The models were performing well, with the area under the curves (AUCs) close to 1 and RF was the best model for the training set and the test set (Supplementary Figures 5A–C and Supplementary Table 2).

**TABLE 2:** Statistically significant compounds (X vs. H). *P*-value < 0.05 (Wilcoxon-Mann-Whitney test). Fold change ≥1.5 or ≤1/1.5. AUC (area under the curve) value of predictors selected by machine learning analysis (see Figure 1, Supplementary Figures 4,5 A–C and Supplementary Tables 1, 2).

Compounds	Fold change	log2fc	*p*-Value	log10p	AUC
DAG36:4(18:1/18:3)	4.111	2.040	6.72E-16	15.173	0.966301
C18:2	13.243	3.727	4.31E-15	14.366	0.956113
C18:3	7.886	2.979	2.15E-14	13.667	0.934169
C18:1	7.888	2.980	7.86E-14	13.105	0.922414
13-HOTrE	2.978	1.575	3.45E-10	9.462	0.880094
9-HODE	2.290	1.195	1.72E-08	7.765	0.85181
13-HODE	3.180	1.669	1.52E-08	7.817	0.846395
9,10-DiHOME	3.235	1.694	1.62E-08	7.791	
9-HOTrE	2.451	1.293	7.28E-07	6.138	
9-OxoODE	1.576	0.657	4.90E-07	6.310	
9-OxoOTrE	1.653	0.725	1.33E-05	4.878	

Among these, 9- and 13-oxylipins, the C18 unsaturated fatty acids, and the DAG36:4 (18:1/18:3) had the most evident fold change in the X samples. The results showed the increase in 9-LOX and 13-LOX oxylipins, together with the increase in C18:1, C18:2, and DAG36:4 (18:1/18:3). These results were consistent with HPLC/MS-MS-targeted analysis performed on olive pools (120 adult trees of Ogliarola salentina naturally infected or not with *Xf)* from Salento in 2017 (Scala et al., 2020).

#### Xf-Positive vs. Xf-Negative in Ogliarola (OX vs. OH) Comparison Highlighted 13-Oxylipins

The 13-oxylipins resulted as the most discriminant entities instead of DAG (check for comparison Table 3 and Supplementary Figure 6). The chosen features for machine learning are indicated in Supplementary Figure 7 and Supplementary Table 3. The model was validated through 10-fold-cross-validation for five times, and they all were performing well, with AUCs = 1 for SVM and NNet, which outperformed RF, having anyway an AUC close to 1 (Supplementary Figures 8A–C). The importance of the selected features in *Xf*-positive/*Xf*-negative classification task was confirmed by applying the trained models on the test set with a default threshold of 0.5. The metrics were excellent for all models, with a sensitivity = 1 and a balanced accuracy of 0.94 (Supplementary Table 1).

**TABLE 3:** Statistically significant compounds (OX vs. OH). *p*-Value < 0.05 (Wilcoxon-Mann-Whitney test). Fold change ≥1.5 or ≤1/1.5. AUC (area under the curve) value of predictors selected by machine learning analysis (see Supplementary Figures 6–8 A–C and Supplementary Tables 1, 3).

Compounds	Fold change	log2fc	*p*-value	log10p	AUC
13-HODE	3.517399	1.814509	7.86E-14	13.104845	1
13-HOTrE	3.483829	1.800674	1.03E-13	12.9886274	1
DAG36:4 (18:1/18:3)	5.120636	2.356323	3.30E-15	14.4820751	1
C18:1	10.550776	3.399277	4.50E-14	13.3467288	0.994048
C18:2	14.746962	3.882346	2.83E-13	12.5480291	0.994048
C18:3	9.806813	3.293784	1.33E-13	12.87454	0.994048
DAG36:4 (18:2/18:2)	1.576154	0.656408	1.34E-14	13.873581	0.994048
DAG36:3 (18:2/18:1)	1.686752	0.754248	5.96E-14	13.2246473	0.991071
9-HODE	2.040263	1.028755	3.35E-09	8.47536291	0.979167
9,10-DiHOME	3.656860	1.870605	0.000182	3.73997161	
9-HOTrE	2.438763	1.286149	2.59E-06	5.58742431	
9-OxoOTrE	1.620215	0.696185	0.005434	2.26487146	
C16:1	1.597930	0.676205	5.95E-05	4.22537613	

#### *Xf*-Positive vs. *Xf*-Negative in Leccino (LX vs. LH) Comparison Highlighted 13-Oxylipins, C18 Mono- and Polyunsaturated Fatty Acid, and DAG

The univariate analysis for the LX vs. LH comparison revealed pretty the same results as X vs. H and OX vs. OH comparison (Table 4 and Supplementary Figure 9). The chosen features for machine learning are indicated in Supplementary Figure 10 and Supplementary Table 4. RF and SVM-trained models were perfect classifiers, outperforming NNet, which was close to the perfect classifier (Supplementary Figures 11A–C). Nevertheless, these models had performed worse on the test set, with a default threshold of 0.5, if compared with the previously analyzed comparisons (Supplementary Table 1).

**TABLE 4:** Statistically significant compounds (LX vs. LH). *P*-value < 0.05 (Wilcoxon-Mann-Whitney test). Fold change ≥1.5 or ≤1/1.5. AUC (area under the curve) value of predictors selected by machine learning analysis (see Supplementary Figures 9–11 A–C and Supplementary Tables 1, 4).

Compounds	Fold change	log2fc	*p*-value	colog10p	AUC
DAG36:4 (18:1/18:3)	3.233282	1.692999	1.62E-06	5.78981954	1
C18:2	11.613076	3.537678	4.42E-07	6.35451662	0.993333
C18:3	6.468369	2.693402	2.89E-05	4.53927894	0.933333
13-HODE	3.132053	1.647109	1.70E-07	6.76949541	0.926667
C18:1	6.159419	2.622794	0.000168	3.77516304	0.88
9-OxoOTrE	1.697533	0.763439	0.000782	3.10683115	0.786667
9-HOTrE	2.692495	1.428944	0.00032	3.49479644	0.776667
13-HOTrE	2.519389	1.333074	0.006039	2.21901933	
9,10-DiHOME	2.823605	1.497538	0.000168	3.77516304	
9-HODE	2.465888	1.302107	0.006613	2.17958842	
9-OxoODE	1.749028	0.806554	0.013275	1.87697959	

#### Ogliarola vs. Leccino (O vs. L) Discriminated by 13-Oxylipins and C18 Polyunsaturated Fatty Acids

The statistically significant compounds (Table 5 and Supplementary Figure 12) were 9-LOX- and 13-LOX-derived oxylipins (9-HOTrE, 13-HODE) and free fatty acids (C18:0, C18:1, C18:2, C18:3). Oxylipins had a positive fold change, while fatty acids had a negative one. 13-HODE seemed to be the lipidic discriminant between the two cultivars. The chosen features for machine learning are shown in Supplementary Figure 13 and Supplementary Table 5. The models were performing well, with AUCs > 0.9 (Supplementary Figures 14A–C). RF was the best model for the training set and performed very well also on test set, with a default threshold of 0.5, but SVM and NNet outperformed RF on the test set, with NNet being close to the perfect classifier (specificity = 0.96, sensitivity = 1, precision = 1, balanced accuracy = 0.98) (Supplementary Table 1).

**TABLE 5:** Statistically significant compounds (O vs., L). *P*-value < 0.05 (Wilcoxon-Mann-Whitney test). Fold change ≥1.5 or ≤1/1.5. AUC (area under the curve) value of predictors selected by machine learning analysis (see Supplementary Figures 12–14 A–C and Supplementary Tables 1, 5).

Compounds	Fold change	log2fc	*p*-value	Colog10p	AUC
13-HODE	2.831960	1.501801	2.93E-13	12.5337425	0.92125
C18:2	0.653510	–0.613718	0.036505	1.43764782	0.547188
C18:3	0.594873	–0.749347	0.020159	1.69552099	0.540313

#### Ogliarola vs. Leccino Xf-Positives (OX vs. LX) Discriminated by 13-HODE

The statistically significant compound (Table 6 and Supplementary Figure 15) was 13-HODE. Other compounds, such as 9-oxylipins (e.g., 9-HOTrE) and P/UFA, had a fold change and a statistical significance near the range imposed. Nevertheless, these and other compounds were the chosen features by machine learning as reported also in Supplementary Figure 16 and Supplementary Table 6. The models were performing well with AUCs close to 1 (Supplementary Figures 17A–C). SVM was the best model for the training set, while NNet was the best on the test set (Supplementary Table 1).

**TABLE 6:** Statistically significant compounds (OX vs. LX). *p*-Value < 0.05 (Wilcoxon-Mann-Whitney test). Fold change ≥1.5 or ≤1/1.5. AUC (area under the curve) value of predictors selected by machine learning analysis (see Supplementary Figures 15–17 A–C and Supplementary Tables 1, 6).

Compounds	Fold change	log2fc	*p*-value	Colog10p	AUC
13-HODE	3.019481	1.594301	6.08E-14	13.2160983	0.982143

#### Ogliarola vs. Leccino *Xf*-Negatives (OH vs. LH) Discriminated by 9- and 13-Oxylipins and Reduction of C18 Polyunsaturated Fatty Acids

The statistically significant compounds (Table 7 and Supplementary Figure 18) were 9-LOX- and 13-LOX-derived oxylipins (9-HOTrE, 13-HODE), free fatty acids (C18:1, C18:2, C18:3), and diacylglycerols DAG36:3(18:2/18:1), DAG36:4(18:1/18:3), DAG36:4(18:2/18:2), and DAG36:2(18:1/18:1). Anyway, as for O vs. L and OX vs. LX, also in OH vs. LH comparison DAGs had few relevant folds change and only oxylipins had a fold change >1.5. This was true for 13-HODE considering all cultivar comparisons, while for 9-HOTrE this was true only in the OH vs. LH comparison. The chosen features for machine learning were C16:1, C18:1, C18:3, 9-HOTrE, 13-HODE, 10-HpOME, DAG36:2(18:2/18:0), DAG36:3(18:2/18:1), DAG36:4(18:1/18:3), and DAG36:4(18:2/18:2) as reported also in Supplementary Figure 19 and Supplementary Table 7. RF was very close to the perfect classifier upon training test (Supplementary Figures 20A–C), but SVM and NNet were the perfect classifiers on the test set (Supplementary Table 1).

**TABLE 7:** Statistically significant compounds (OH vs. LH). *p*-Value < 0.05 (Wilcoxon-Mann-Whitney test). Fold change ≥1.5 or ≤1/1.5. AUC (area under the curve) value of predictors selected by machine learning analysis (see Supplementary Figures 18–20 A–C and Supplementary Tables 1, 7).

Compounds	Fold change	log2fc	*p*-value	Colog10p	AUC
13-HODE	2.688685	1.426901	2.74E-10	9.56276094	1
9-HOTrE	1.622176	0.697931	2.74E-10	9.56276094	1
C18:1	0.442457	–1.176392	0.001028	2.98812671	0.916667
C18:2	0.558336	–0.840796	0.012294	1.91032049	0.908333
C18:3	0.428582	–1.222357	0.000186	3.72953922	0.883333

#### Dentamet^®^ Treated vs. Untreated *Xf*-Positives (DX vs. NDX) Discriminated by Modulation of Lipoxygenase and Dioxygenase Oxylipins

The statistically significant compounds (Table 8 and Supplementary Figure 21) were 9-LOX- and 13-LOX-derived oxylipins (9-OxoODE, 9-HOTrE,13HOTrE, 12,13-DiHOME), free fatty acids (C16:0, C18:0, C18:1), 10DOX-derived oxylipins (10-HpOME), monoacylglycerols (MAG18:0), and diacylglycerols [DAG36:3(18:2/18:1)]. Only oxylipins had a fold change ≥1.5 or ≤1.5. Precisely, 9-OxoODE and 9-HOTrE were positively increased, while 13-HODE, 13-HOTrE, 12,13-DiHOME, and 10-HpOME were decreased. The decrease in 10-HpOME could be a cue of Dentamet^®^ action against *Xf* vitality. As well as the decrease in 13-HODE and 13-HOTrE and the increase in 9-HOTrE, that is, the LOX-derived oxylipins. The alteration (↓10-HpOME; ↓13-HOD/TrE; ↑9-HOD/TrE) of these compounds in the Dentamet^®^-treated samples could be correlated with the beneficial effects of this treatment. The chosen features for machine learning were C16:0, C18:0, C18:1, 9-OxoODE, 13-HODE, 13-HOTrE,12,13-DiHOME, 10-HpOME, DAG36:3(18:2/18:1), and DAG36:4(18:2/18:2) as indicated in Supplementary Figure 22 and Supplementary Table 8. RF and NNet were very close to the perfect classifier upon training test (Supplementary Figures 23A–C), and they were the perfect classifier on the test set (Supplementary Table 1).

**TABLE 8:** Statistically significant compounds (DX vs. NDX). *P*-value < 0.05 (Wilcoxon-Mann-Whitney test). Fold change ≥1.5 or ≤1/1.5. AUC (area under the curve) value of predictors selected by machine learning analysis (see Supplementary Figures 21–23 A–C and Supplementary Tables 1, 8).

Compounds	Fold change	log2fc	*p*-value	Colog10p	AUC
12,13-DiHOME	0.575100	–0.798116	1.88E-13	12.725417	0.914352
DAG36:4 (18:1/18:3)	1.149619	0.201155	0.006161	2.210325	0.912616
DAG36:3 (18:2/18:1)	1.180973	0.239975	1.25E-12	11.902883	0.912616
13-HOTrE	0.7022324	–0.509980	0.022419	1.6493758	0.810185
10-HpOME	0.454825	–1.136617	2.49E-07	6.6034695	0.769676
9-OxoODE	1.587522	0.666776	0.000122	3.9136477	0.768519
C18:0	0.705027	–0.504250	8.52E-07	6.0693574	0.744213
C16:0	0.729496	–0.455027	0.000619	3.2079756	0.726852
13-HODE	0.522108985	–0.937577	0.009958	2.001846	0.616898
C18:1	1.314170	0.394152	0.03267	1.4858567	0.607639
9-HOTrE	2.146936	1.102279	5.81E-06	5.2359543	
9,10-DiHOME	0.694181	–0.526616	0.03451	1.4620545	
MAG18:0	0.994803	–0.007517	0.00024	3.6202988	

### Bioinformatic Analysis of RNAseq Data Files

**FIGURE 2:**
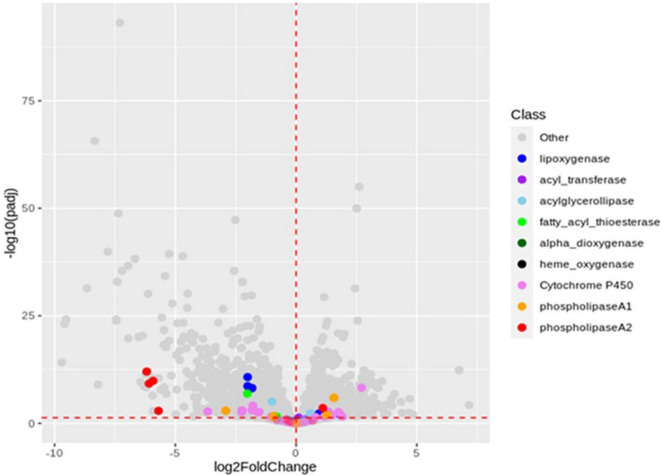
Volcano plot of X vs. H comparison. X-axis: log_2_ of genes fold change. Y-axis: −log_10_ of the adjusted *p*-value. The vertical red dashed line is set on *x* = 0, separating downregulated genes (left side) from upregulated (right side). The horizontal red dashed line is set on *y* = −log_10_0.05, as a threshold for statistically significant genes. The gene names reported in the volcano plot are filtrated from the RNA-seq data available in the *de novo.cnag.cat Olea europea* directory (https://denovo.cnag.cat/olive_data?fid~=~161#block-likable-page-title). The annotation is carried out by gene ontology guidelines as reported in Giampetruzzi et al., 2016. Starting from the protein annotation dataset, we design a subset by functional category to find gene’s transcripts involved in the fatty acid metabolism, including the hypothetical ones.

For bioinformatic analysis, we used RNAseq files from the published study (Giampetruzzi et al., 2016), available online(see text footnote 1). The authors performed a global transcriptomic analysis to assess the differences between cultivars (Ogliarola salentina and Leccino) and between *Xf-*positive and -negative samples (Giampetruzzi et al., 2016). Starting from these files, we performed a targeted transcriptomic analysis focusing on the lipid metabolism-related genes. Several comparisons were assessed among the samples, namely, X vs. H, OX vs. OH, LX vs. LH, O vs. L, OX vs. LX, and OH vs. LH. The analysis revealed that, in the X vs. H comparison, the lipid genes pathways of the olive samples appeared severely altered in consequence of *Xf* presence. In particular, (1) acyltransferases, putatively involved in the synthesis of phosphatidic acid (PA, a well-known plant defense modulator); (2) phospholipases, active in providing free fatty acids and other signal molecules (such as PA and glycerol-3-phosphate; Siebers et al., 2016); and (3) lipoxygenases, active in transforming PUFA into antimicrobial or hormonal molecules (such as jasmonates; Wasternack, 2007; Figure 2). The analysis of the OX vs. LX comparison revealed at least two 13-LOX upregulated and two 9-LOX downregulated in Ogliarola salentina compared to Leccino (Supplementary Table 9 and Supplementary Figure 24).

## Discussion

Previous studies (Scala et al., 2020) compared *Xf*-positive and *Xf*-negative olive sample pools, identifying putative infection biomarkers in cv Ogliarola salentina: the compounds were oleic/linoleic/linolenic acid-deriving oxylipins [(9-HODE; 9-HOTrE; 13-HODE; 13-HOTrE; 13-oxo-octadecenoic acid (13-oxoODE); 10-hydroxyoctadecenoic acid (10-HODE); 10-hydroperoxyoctamonoenoic acid (10-HpOME)], unsaturated fatty acids (oleic acid—C18:1; linoleic acid—C18:2); and diacylglycerol [DAG36:4 (18:1/18:3)]. Oleic acid is among the modulators of quorum sensing in *Xf* while DAG-associated compounds (e.g., DAG 36:2) establish an appropriate defense response by inducing defense-signaling molecules (Siebers et al., 2016; Scala et al., 2020). Oleic acid-derived DOX-oxylipins have been reported to moderate the lifestyle of *P. aeruginosa* (Martínez and Campos-Gómez, 2016), and to affect *in vitro* biofilm formation in *Xf* (Scala et al., 2020). As regards LOX-derived products, 9- and 13-oxylipins proved to modulate biofilm formation *in vitro* (Scala et al., 2020), at least at the tested concentrations (8 μM to 0.8 mM).

With this study we aim to search for the lipid determinants related to *Xf* infection, olive tree cvs [Ogliarola salentina (O)—susceptible to OQDS and Leccino (L)—resistant to OQDS] and treatment with Dentamet^®^ under open field conditions. Regarding this, we study a big dataset represented by 66 olive trees (Table 1) belonging to the two cultivars, which are healthy (H), naturally infected by *Xf* (X), and, specifically to this latter, treated or not with Dentamet^®^ (DX; Scortichini et al., 2018; Tatulli et al., 2021). RF, SVM, and NNet are applied successfully in lipidomic data analysis to build robust models for infection, cultivar, and treatment discrimination and biomarker selection. 9- and 13-LOX-derived oxylipins, free fatty acids (C16:1, C18:1, C18:2, C18:3), and some diacylglycerols [especially DAG36:4(18:1/18:3)] are revealed as predictors of the different thesis.

**FIGURE 3:**
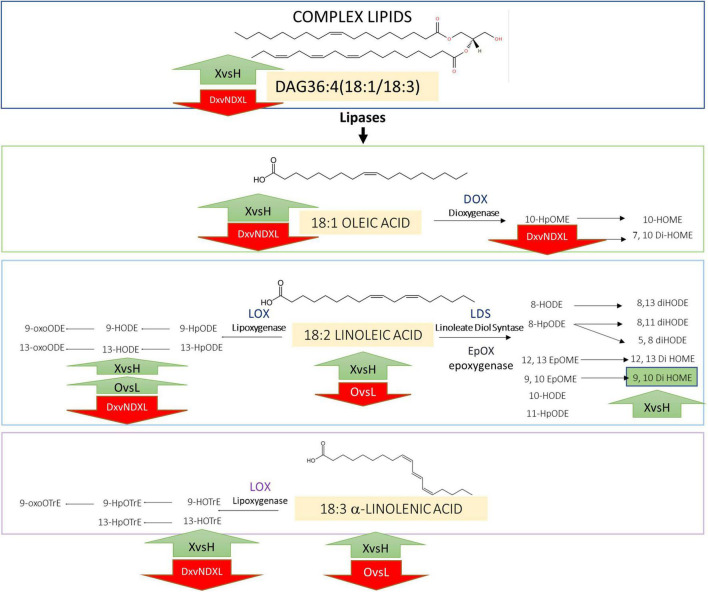
Proposed outline of the lipid metabolism in the two cultivars Ogliarola salentina (O) and Leccino (L) in healthy (H) and in *X. fastidiosa*-infected (X) olive trees; these latter treated (DX) or untreated (NDX) with Dentamet^®^. Complex lipids (e.g., diacylglycerides) cleaved by lipases produce free fatty acids (e.g., oleic/linoleic/linolenic acids) that, in turn, are oxidized by enzymes like lipoxygenases (LOX) and dioxygenases (DOX; LDS) and epoxygenases (EpOX) to produce oxylipins [e.g., 9-hydroxyoctadecenoic acid (9-HODE) and 10-hydroperoxyoctamonoenoic acid (10HpOME)].

In olive trees, we provided a scenario of lipid pathways leading to the formation of oxylipins during the infection in the Ogliarola salentina (Scala et al., 2020). This study confirms the importance of these lipid pathways using a new set of trials including (Leccino) and treatment able to alleviate OQDS. The comparison of the dataset of infected (X) vs. non-infected (H) olive trees confirms biomarkers of the *X. fastidiosa* infection and suggests their position in the complex lipid pathway as illustrated in Figure 3. Complex lipids, such as diacylglycerides, appear crucial in several pathosystems for the onset of plant defenses (Siebers et al., 2016). DAG36:4(18:1, 18:3) is induced by *Xf* (up to approximately fivefolds). Complex lipids provide a substrate for lipases/esterases to produce active compounds, such as oxylipins and hormones. LesA, a lipase/esterase produced by different subspecies of *Xf*, is among the most secreted virulence factor during the interaction with the hosts (Nascimento et al., 2016; de Souza et al., 2020). The bioinformatic analysis (Figure 2) on the RNAseq data of Giampetruzzi et al. (2016) indicates that several lipases are differentially expressed in olive trees infected by *Xf*. This result suggests that in X vs. H the increase in C18:1, C18:2, and C18:3 could derive from DAGs and by lipases activity (Figures 2, 3). These free fatty acids are active molecules in plant defenses, and C18:1 modulates the expression of genes with antivirulence activity in several bacteria (Chowdhury et al., 2021). C18:2 possesses an antibiofilm effect in humans’ pathogenic bacteria (Lee et al., 2017) while C18:3 is the main substrate for the synthesis of the defense-hormones jasmonates relevant even in Pierce’s disease (Choi et al., 2013; Siebers et al., 2016). The accumulation of these P/UFA appears related to the defense reaction of olive trees against *Xf*. Nonetheless, oleic, linolenic, and linolenic acids are substrates for the synthesis of hormone-like oxylipins. These compounds have already been recognized as crucial in controlling the lifestyle of *P. aeruginosa* (Martínez and Campos-Gómez, 2016). *Xf* infection triggers the expression of several fatty acid oxidases in olive trees, that is LOX, DOX, and CYP450 monooxygenases (Giampetruzzi et al., 2016). Oxylipins derived from the LOX pathway, such as 9/13-HOD/TrE, and from epoxygenases, such as 9,10 diHOME, are triggered in OQDS trees (Figures 1, 3) while others are depressed (i.e., 9-oxoOD/TrE). This picture is a sort of “oxylipins signature” of the infection. The oxylipins that increase during infection (Table 2) could affect *Xylella* lifestyle (i.e., promoter of biofilm), have antibacterial activity (Mundt et al., 2003), and promote oxidative stress (9,10-di HOME; Gabbs et al., 2015). The oxylipins that decrease in the infected samples (e.g., 9-oxoODE and 9-oxoOTrE) are plant growth promoters and enhancers of resistance to multiple stresses (Ihara et al., 2021; Table 2 and Figure 3).

Focusing on the Ogliarola salentina versus Leccino comparisons (O vs. L or OX vs. LX or OH vs. LH), other lipid entities discriminate the two cultivars, namely, the different accumulation of LOX-oxylipins and of P/UFA. We can speculate that the higher degree of 13-oxylipins with *Xf* infection appears as varietal traits of Ogliarola salentina at least in comparison with Leccino (Table 6). The two varieties differ even in the healthy status; specifically, 13-HODE and 9-HOTrE are higher, whereas P/UFA are lower in OH vs. LH (Table 7). *Xf* could find a “lipid profile” in healthy trees that support susceptibility of Ogliarola salentina compared to Leccino. Probably, these lipids are essential in modulating the occurrence of OQDS symptoms.

After *Xf* infection, 9-LOX-derived oxylipins do not significantly differentiate the two cultivars (Supplementary Figure 15) while 13-oxylipins are confirmed as *Xf* infection biomarker of Ogliarola salentina. Bioinformatic analysis of RNAseq data of Giampetruzzi et al. (2016) supports this result, namely, at least two hypothetical 9-*lox* are found downregulated and two 13-*lox* upregulated in *Xf*-infected samples, that is, OX vs. LX. Vellosillo et al. (2007) found that the inactivation of the 9-LOX signaling pathway augmented bacterial growth and reduced the activation of the salicylic acid-inducible defense genes in *Arabidopsis* infected with *P. syringae* pv. *tomato* (Pst) DC3000. In the same pathosystem, Vicente et al. (2012) demonstrated that 9-LOX derivatives promote plant defenses-reducing bacterial symptoms. The role of the 13-HODE is trickier to explain since few information on its role in the plant-bacteria interaction is available. This oxylipin in mammals has a cell proliferative effect and an antibacterial activity against human bacterial pathogens, such as *Staphylococcus aureus* (Mundt et al., 2003). Moreover, in our previous study, the 13-HODE behaves as a promoter of biofilming in *Xf* (Scala et al., 2020).

The results of the lipidomic analysis of the DX samples suggest that Dentamet^®^ can reshape lipid profile in the *Xf*-infected olive trees. Besides, Dentamet^®^ reduces the load of *Xf* into infected samples and OQDS symptoms (Tatulli et al., 2021). Dentamet’s effect results are evident in the decrease in 13-HODE, 13-HOTrE (putatively of plant origin) and 10-HpOME (probably of bacterial origin), and an increase in 9-HOTrE. Thus, we can figure out that Dentamet^®^ reduces the bacterial titer and causes a general reassortment of the oxylipin pathways in olive trees as well as in *Xf*. Even though the decrease in 10-HpOME – a bacterial oxylipin (Martínez and Campos-Gómez, 2016) – could be a cue to explain the action of Dentamet^®^ against *Xf* vitality, less trivial appear the up-/downmodulation of the other LOX-oxylipins. The decrease in 13-LOX and the increase in 9-LOX products in Dentamet^®^-treated samples should be further investigated. The reported beneficial effect of Dentamet^®^ on infected trees could be due, in addition to the bactericidal activity of this compound (Scortichini et al., 2018; Tatulli et al., 2021), to the capability of this treatment to enhance plant defenses *via* 9-oxylipins (Vicente et al., 2012).

Previous metabolomic studies about *Xf*-infected olive trees were focused on secondary metabolites and found azelaic acid (AZA; Nicolì et al., 2019) and quinic acid (Girelli et al., 2019) as *Xf* infection biomarkers. Notably, AZA can be synthesized *via* ROS acting on C18 membrane lipids (Vlot et al., 2021), supporting our findings in the X vs. H comparison that clearly indicate the accumulation of C18.

It is evident that the combinational usage of multiple univariate, multivariate, and machine learning methods may provide more comprehensive information for a global understanding of the metabolomics or other “omics” data. This study suggests that machine learning algorithms could empower the research of biomarkers of different biological systems, combining the precise information from HPLC-MS/MS with fined-tuned prediction models. Owing to the great potential of this approach, prospective studies are needed to broaden its application to a larger scale, maybe switching from HPLC-MS/MS to the faster DI-MS/MS, in combination with molecular techniques, never forgetting that the use of machine learning algorithms is case-sensitive and requires careful evaluation before applying them to a particular task. Moreover, *lox* genes can be factors in modulating olive trees defenses to *Xf* and can be a target for plant-bacteria interaction study in the model organism as *Arabidopsis*.

## Data Availability Statement

The datasets presented in this study can be found in online repositories. The names of the repository/repositories and accession number(s) can be found in the article/Supplementary Material.

## Author Contributions

VS, SL, NP, and MR contributed to conceptualization of the study. MSA, GT, AC, and NP contributed to methodology of the study. VS, MR, MSC, and SL contributed to the investigation of the data. MSA, MR, and VS wrote the original draft. MSC, SL, MR, VS, and NP contributed to writing, reviewing, and editing the manuscript. MR contributed to visualization. MR, MSC, and SL contributed to funding acquisition. MR and VS contributed to supervision. All authors contributed to the article and approved the submitted version.

## Conflict of Interest

The authors declare that the research was conducted in the absence of any commercial or financial relationships that could be construed as a potential conflict of interest.

## Publisher’s Note

All claims expressed in this article are solely those of the authors and do not necessarily represent those of their affiliated organizations, or those of the publisher, the editors and the reviewers. Any product that may be evaluated in this article, or claim that may be made by its manufacturer, is not guaranteed or endorsed by the publisher.

## Footnotes

1
http://trace.ncbi.nlm.nih.gov/Traces/sra/
