# Supplementary material for: Mass Spectrometry-Based Targeted Lipidomics and Supervised Machine Learning Algorithms in Detecting Disease, Cultivar, and Treatment Biomarkers in Xylella fastidiosa subsp. pauca-Infected Olive Trees
Source: Front Plant Sci. 2022 Apr 22;13:833245. doi: 10.3389/fpls.2022.833245 (PMC9072861; doi:10.3389/fpls.2022.833245)
Supplement: Supplementary file 1 [file Data_Sheet_1.PDF]

Supplementary Material

1 Supplementary Figures and Tables

1.1 Supplementary Figures

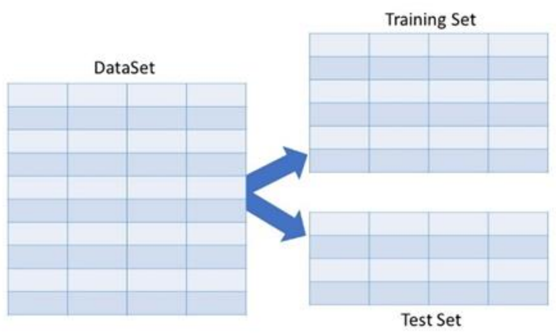

Supplementary Figure 1. Representation of Dataset splitting.

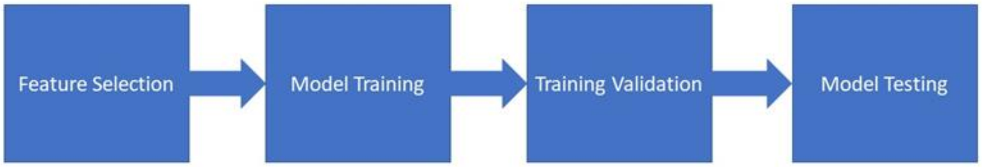

Supplementary Figure 2. Representation of machine learning pipeline.

Sensitivity (True Positive Rate)	TPR = TP/P = TP/(TP+FN)
Specificity (True Negative Rate)	TNR = TN/N = TN/(TN+FP)
Precision (Positive Predicted Value)	PPV= TP/(TP+FP)= 1-FDR
False Discovery Rate	FDR=FP/(FP+TP)=1-PPV
Accuracy	ACC=(TP+TN)/(TP+TN+FP+FN)
Balanced Accuracy	BA=(TPR+TNR)/2

Supplementary Figure 3 Representation of metrics computation.

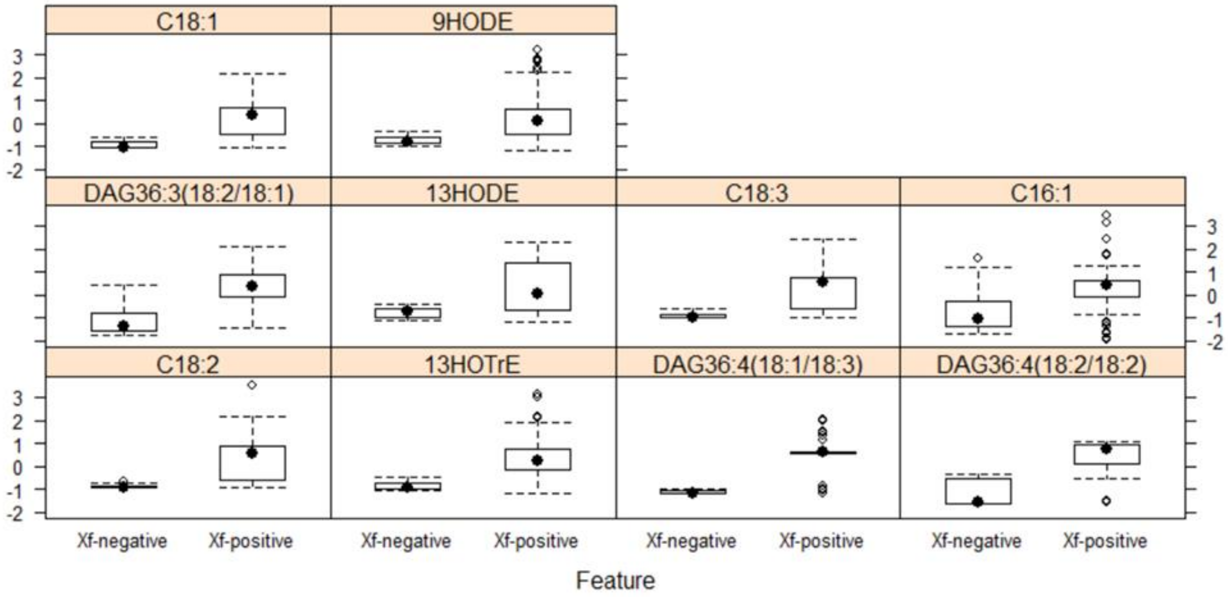

Supplementary Figure 4. *Xf*-infected (X) vs Healthy (H) samples. Machine Learning Analysis.Feature selection: top 10 predictors found by Random Forest algorithm. Values on y-axis represent relatives amount of compounds. These values are peak areas normalize on IS and then centered and scaled before machine learning analysis

a) b) c)

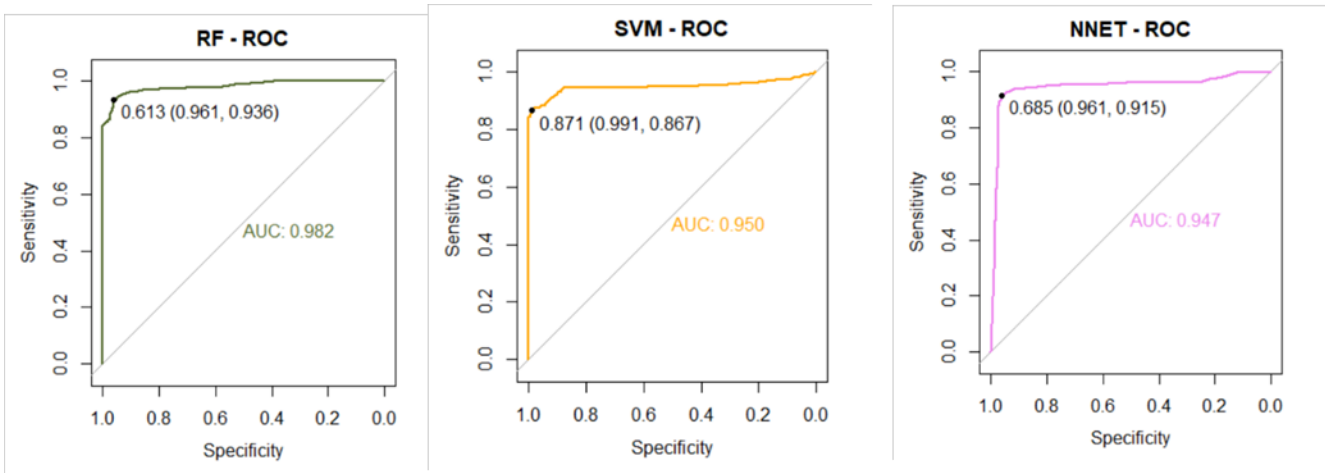

Supplementary Figure 5 a-c. *Xf*-infected (X) versus Healthy (H) samples. ROC (Receiving Operator Characteristics) curves of the trained model applied on training set. The turning slope point represent the best threshold, with the optimal couple of Specificity and Sensitivity as coordinate. AUC is the Area Under Curve.

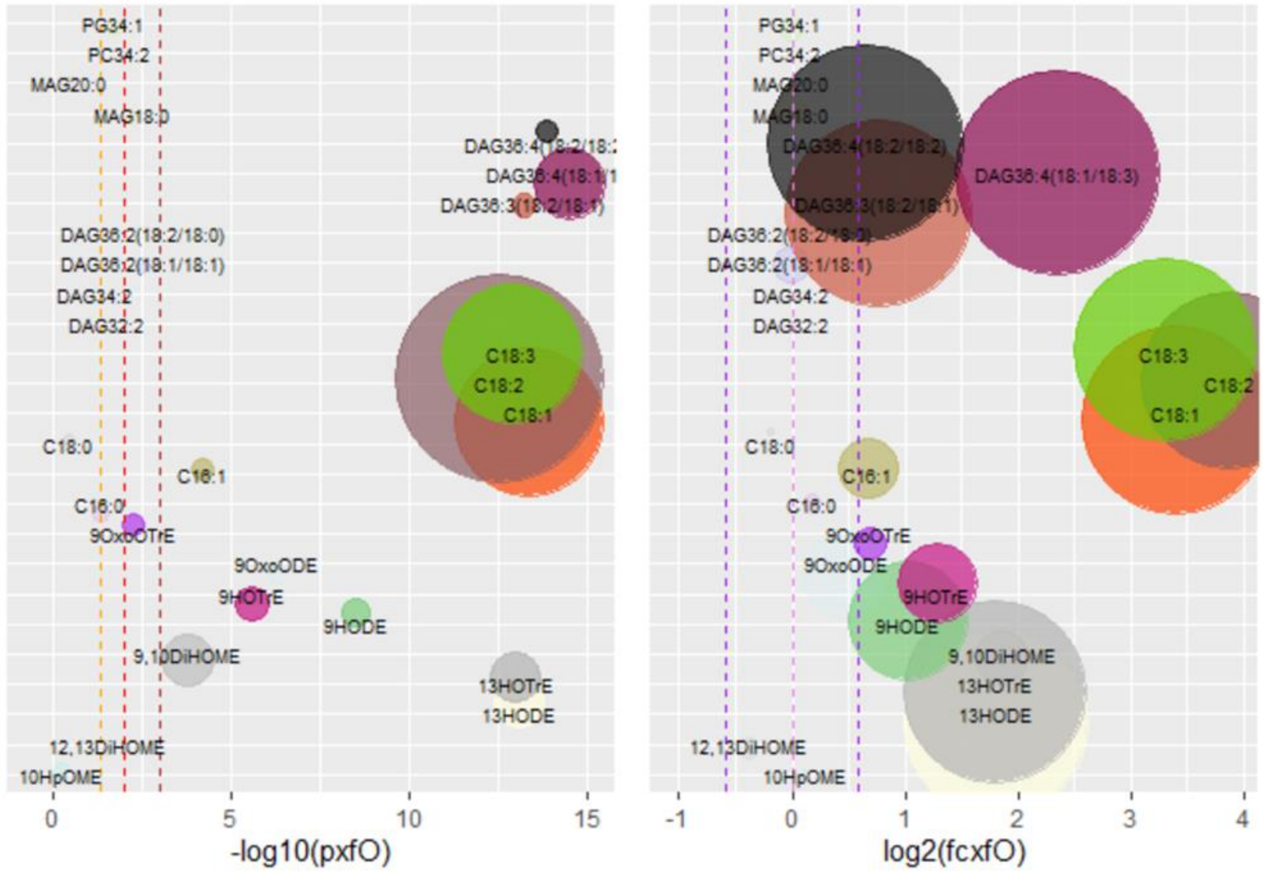

Supplementary Figure 6. *Xf*-infected (OX) versus Healthy (OH) samples in Ogliarola salentina. Univariate statistical analysis. Statistical significance (Wilcoxon Mann Whitney test) and fold change of lipid entities. The left panel shows statistically significant compounds: on x-axis there is the -log10(p-value), on y-axis compounds name. The vertical dashed lines correspond to, respectively, -log10(0.05), -log10(0.01), -log10(0.001). Compounds under the first threshold are represented as transparent, compounds above the threshold are in full colors. Circle dimensions are proportional to fold changes. On right panel x-axis there is instead the log2(fold-change), on y-axis again compounds name. The right panel point out compounds with the biggest fold changes, represented in full colors, while compounds with a fold change>=1.5 or <=1.5 are transparent. Circle dimensions are proportional to -log10(p-value).

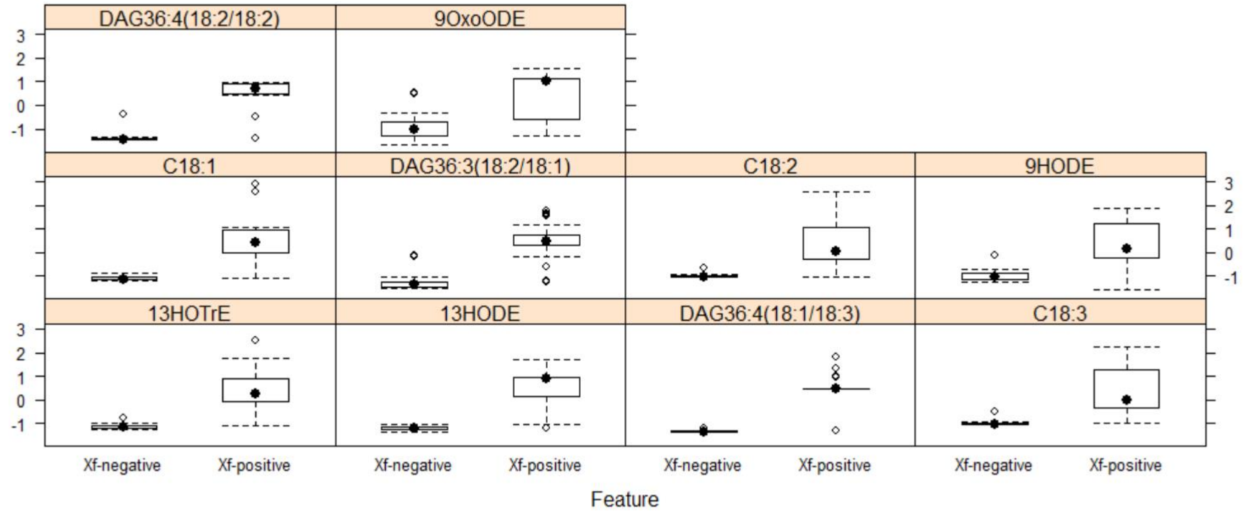

Supplementary Figure 7. *Xf*-infected (OX) versus Healthy (OH) samples in Ogliarola salentina. Machine Learning Analysis. Feature selection: top 10 predictors found by Random Forest algorithm. Values on y-axis represent relatives amount of compounds. These values are peak areas normalize on IS and then centered and scaled before machine learning analysis

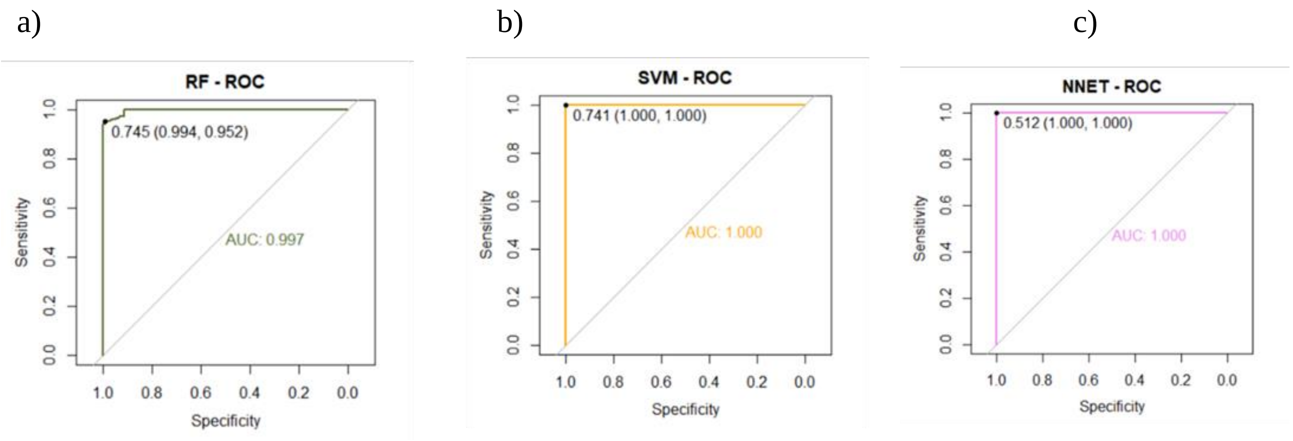

Supplementary Figure 8 a-c. *Xf*-infected (OX) versus Healthy (OH) samples in Ogliarola salentina. ROC (Receiving Operator Characteristics) curves of the trained model applied on training set. The turning slope point represent the best threshold, with the optimal couple of Specificity and Sensitivity as coordinate. AUC is the Area Under Curve.

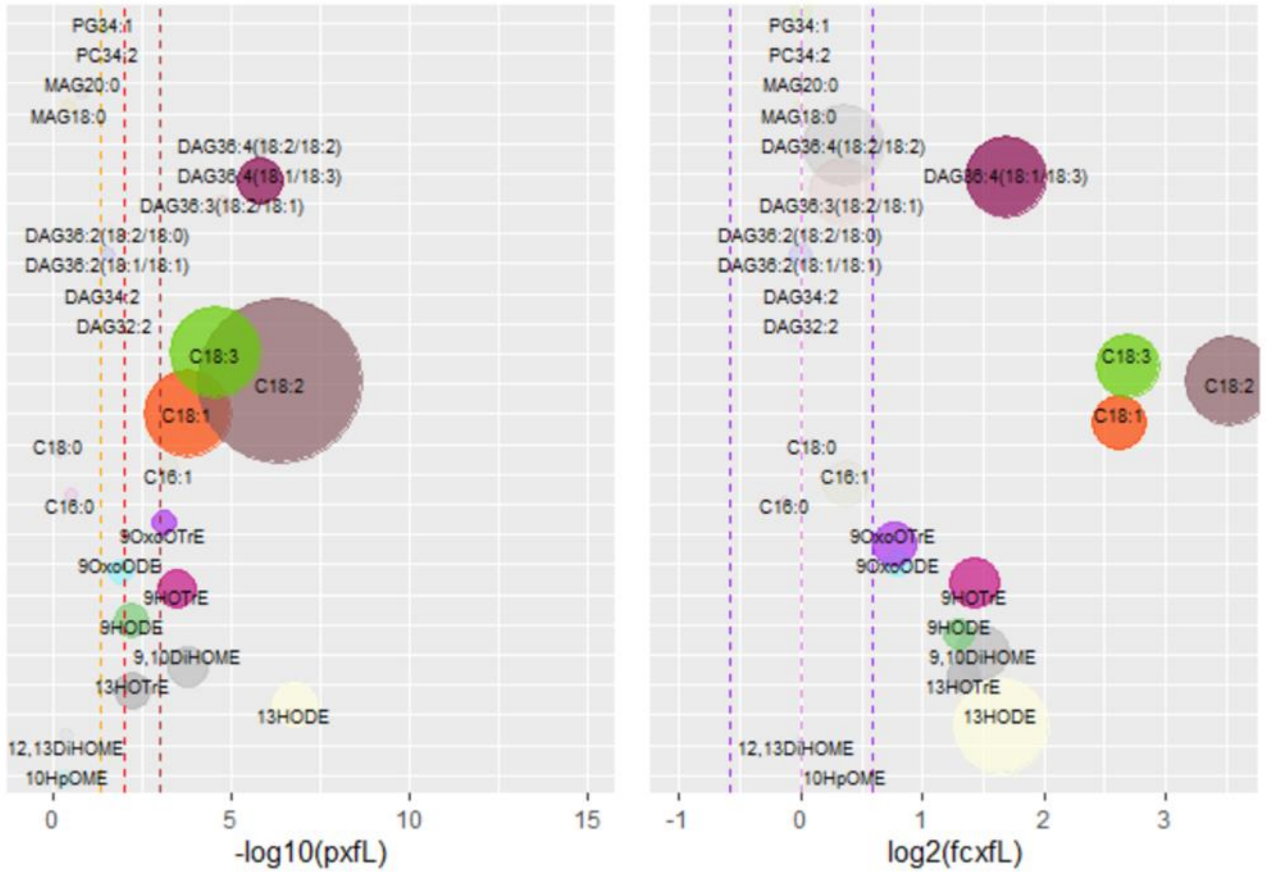

Supplementary Figure 9. *Xf*-infected (LX) versus Healthy (LH) samples in Leccino. Univariate statistical analysis. Statistical significance (Wilcoxon Mann Whitney test) and fold change of lipid entities.The left panel shows statistically significant compounds: on x-axis there is the -log10(p-value), on y-axis compounds name. The vertical dashed lines correspond to, respectively, -log10(0.05), -log10(0.01), -log10(0.001). Compounds under the first threshold are represented as transparent, compounds above the threshold are in full colors. Circle dimensions are proportional to fold changes. On right panel x-axis there is instead the log2(fold-change), on y-axis again compounds name. The right panel point out compounds with the biggest fold changes, represented in full colors, while compounds with a fold change>=1.5 or <=1.5 are transparent. Circle dimension are proportional to -log10(p-value).

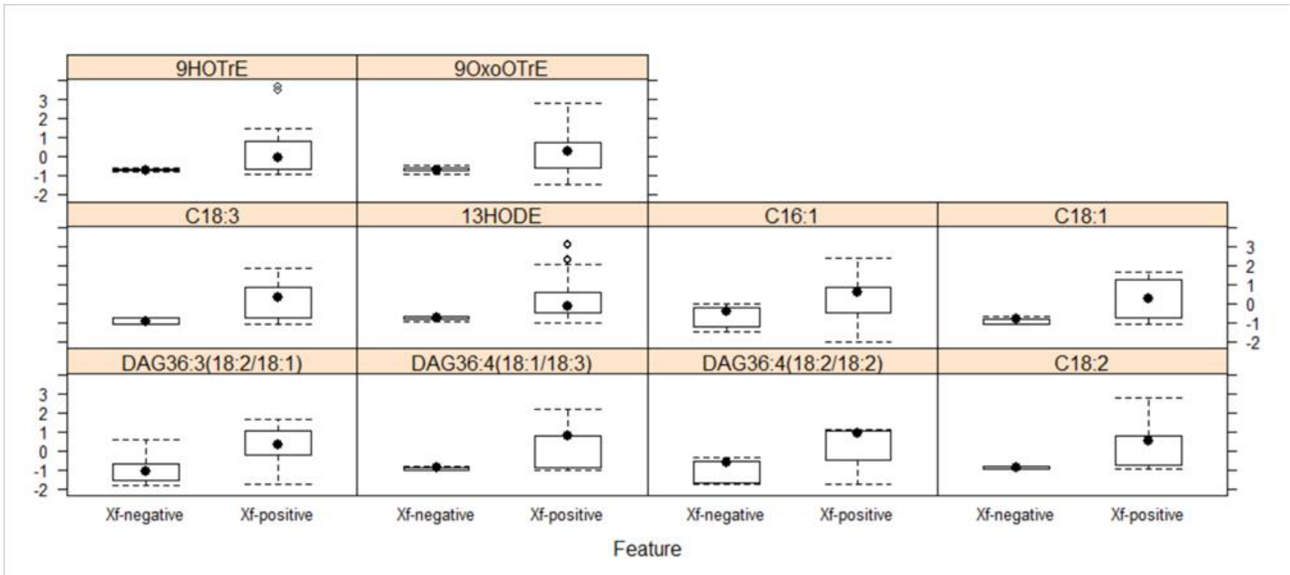

Supplementary Figure 10. *Xf*-infected (LX) versus Healthy (LH) samples in Leccino. Machine Learning Analysis. Feature selection: top 10 predictors found by Random Forest algorithm. Values on y-axis represent relatives amount of compounds. These values are peak areas normalize on IS and then centered and scaled before machine learning analysis

a) b) c)

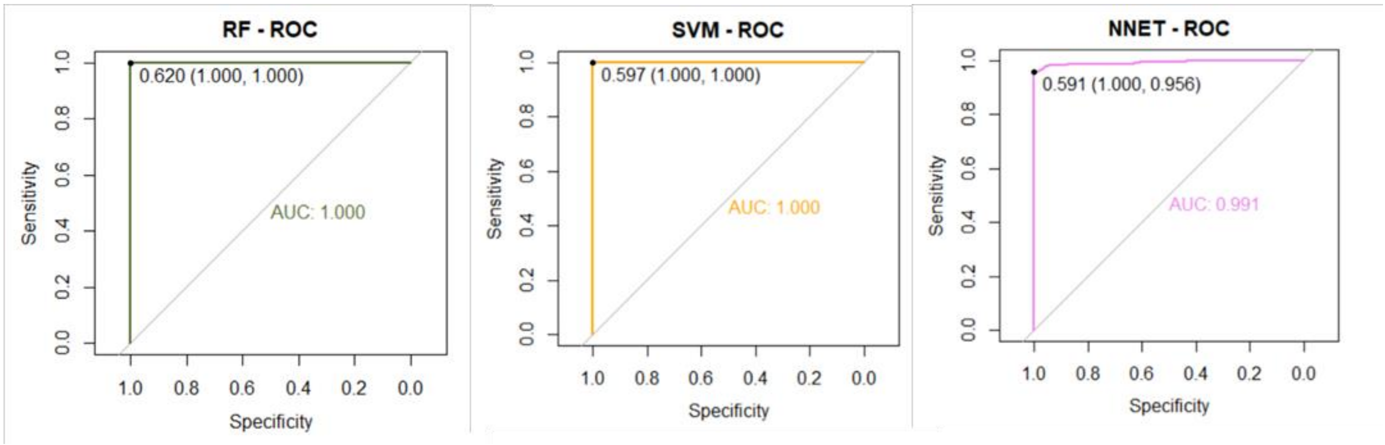

Supplementary Figure 11 a-c. *Xf*-infected (LX) versus Healthy (LH) samples in Leccino. ROC (Receiving Operator Characteristics) curves of the trained model applied on training set. The turning slope point represent the best threshold, with the optimal couple of Specificity and Sensitivity as coordinate. AUC is the Area Under Curve.

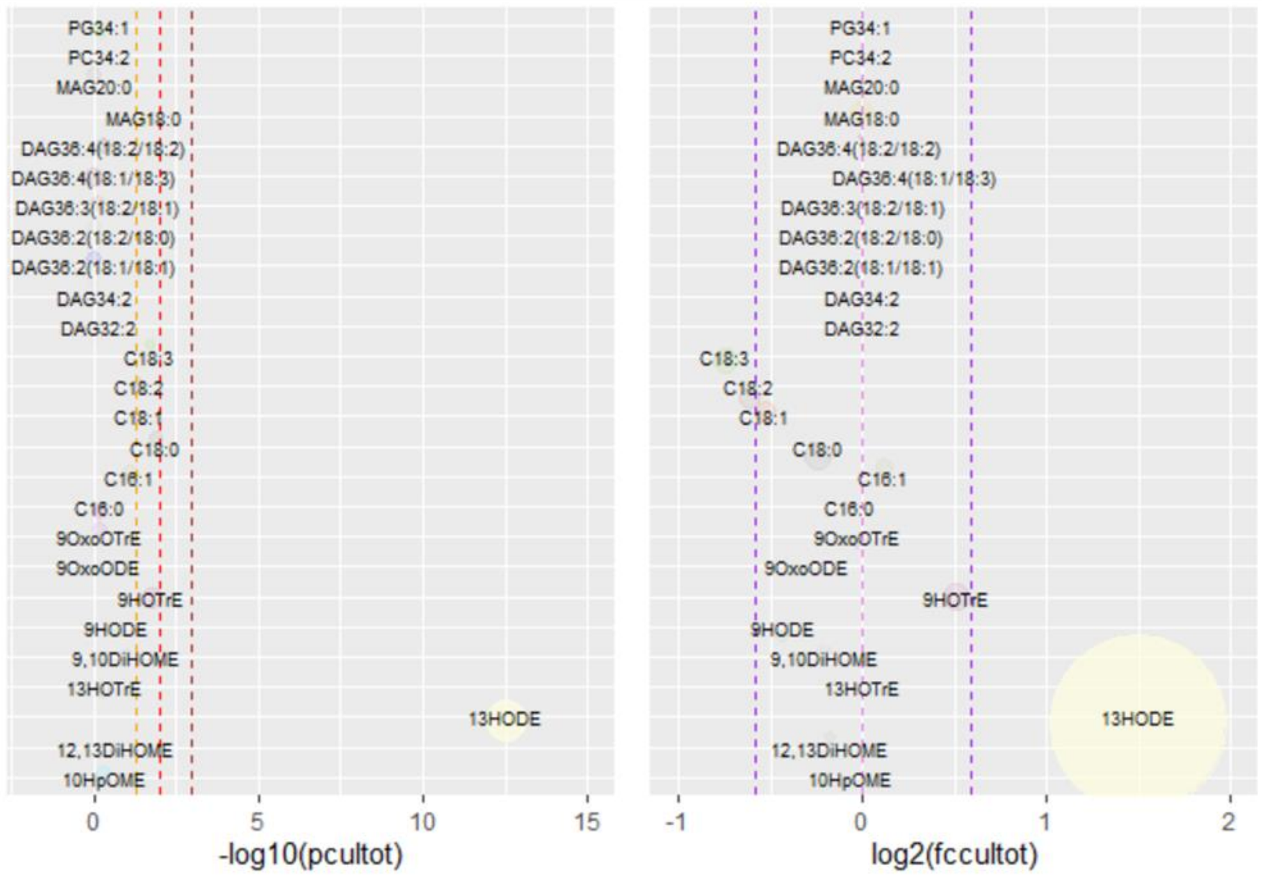

Supplementary Figure 12. Ogliarola salentina (O) vs Leccino (L)**.** Univariate statistical analysis. tatistical significance (Wilcoxon Mann Whitney test) and fold change of lipid entities. The left panel shows statistically significant compounds: on x-axis there is the -log10(p-value), on y-axis compounds name. The vertical dashed lines correspond to, respectively, -log10(0.05), -log10(0.01), -log10(0.001). Compounds under the first threshold are represented as transparent, compounds above the threshold are in full colors. Circle dimensions are proportional to fold changes. On right panel x-axis there is instead the log2(fold-change), on y-axis again compounds name. The right panel point out compounds with the biggest fold changes, represented in full colors, while compounds with a fold change>=1.5 or <=1.5 are transparent. Circle dimension are proportional to -log10(p-value).

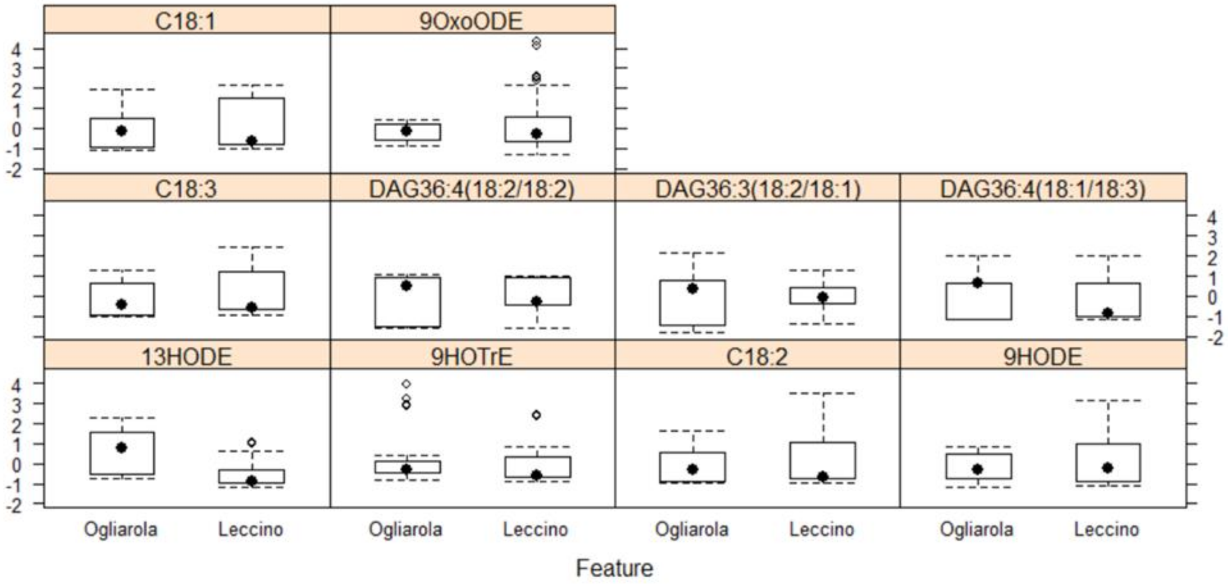

Supplementary Figure 13. Ogliarola salentina (O) vs Leccino (L)**.** Machine Learning Analysis.Feature selection: top 10 predictors found by Random Forest algorithm. Values on y-axis represent relatives amount of compounds. These values are peak areas normalize on IS and then centered and scaled before machine learning analysis

a) b) c)

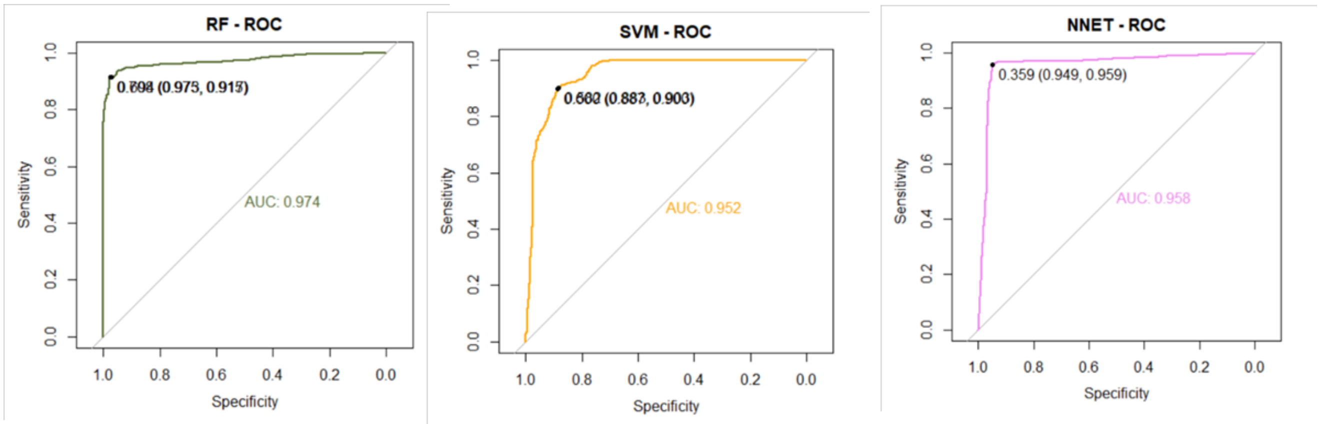

Supplementary Figure 14 a-c. Ogliarola salentina (O) vs Leccino (L)**.** ROC (Receiving Operator Characteristics) curves of the trained model applied on training set. The turning slope point represent the best threshold, with the optimal couple of Specificity and Sensitivity as coordinate. AUC is the Area Under Curve.

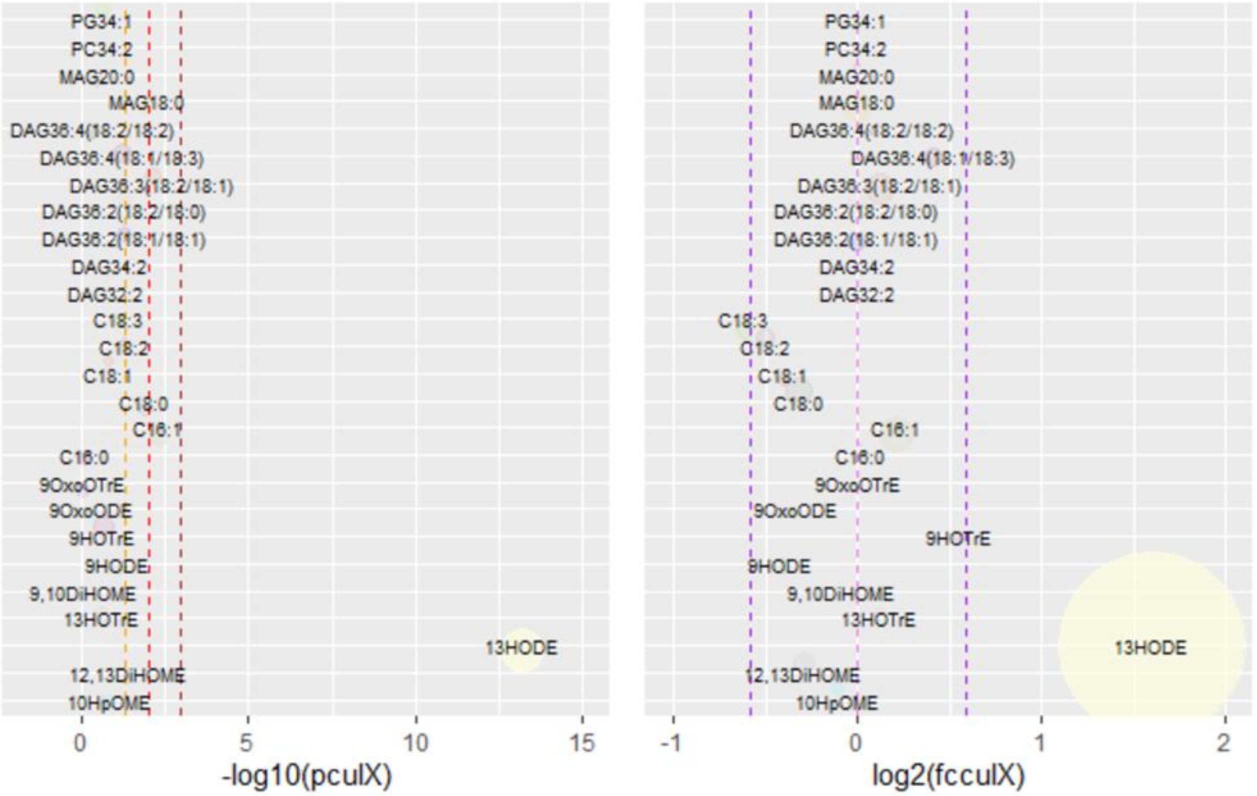

Supplementary Figure 15. Ogliarola salentina (OX) vs Leccino (LX) in *Xf* infected samples. Univariate statistical analysis. Statistical significance (Wilcoxon Mann Whitney test) and fold change of lipid entities. The left panel shows statistically significant compounds: on x-axis there is the -log10(p-value), on y-axis compounds name. The vertical dashed lines correspond to, respectively, -log10(0.05), -log10(0.01), -log10(0.001). Compounds under the first threshold are represented as transparent, compounds above the threshold are in full colors. Circle dimensions are proportional to fold changes. On right panel x-axis there is instead the log2(fold-change), on y-axis again compounds name. The right panel point out compounds with the biggest fold changes, represented in full colors, while compounds with a fold change>=1.5 or <=1.5 are transparent. Circle dimension are proportional to -log10(p-value).

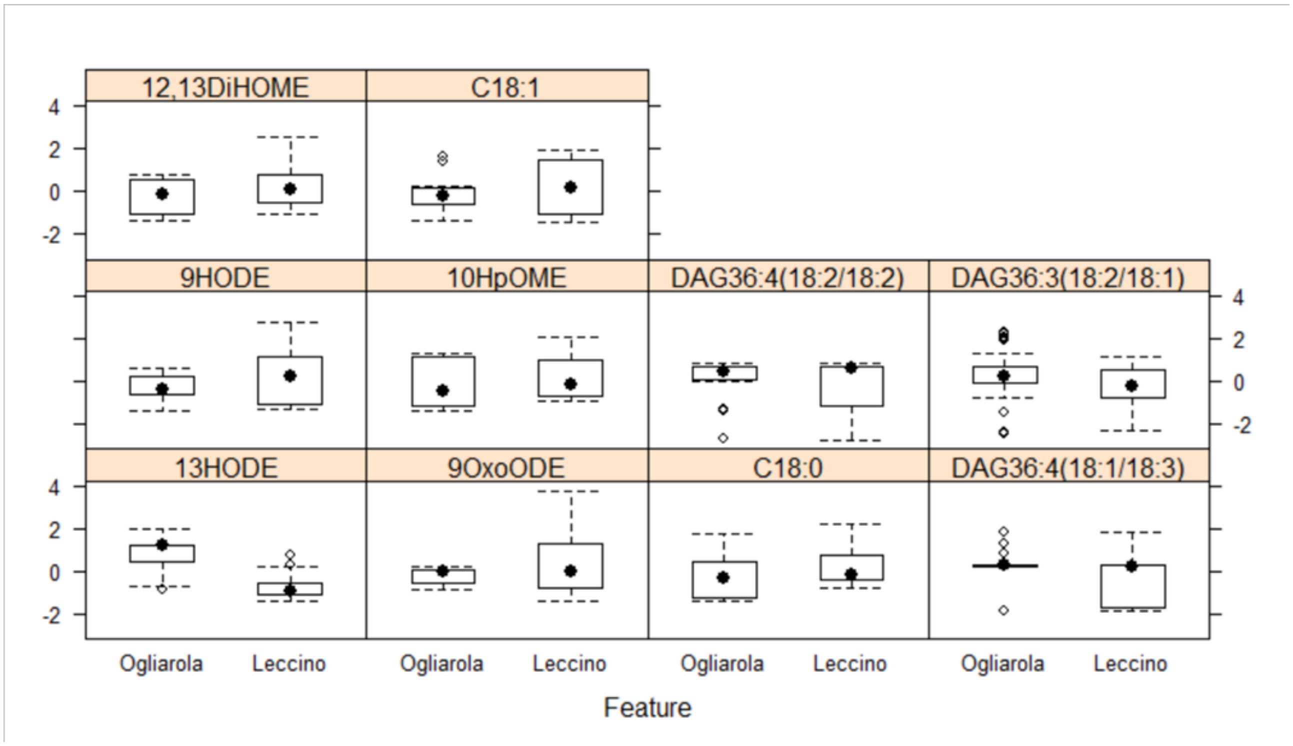

Supplementary Figure 16. Ogliarola salentina (OX) vs Leccino (LX) in *Xf* infected samples. Machine Learning Analysis. Feature selection: top 10 predictors found by Random Forest algorithm. Values on y-axis represent relatives amount of compounds. These values are peak areas normalize on IS and then centered and scaled before machine learning analysis

a) b) c)

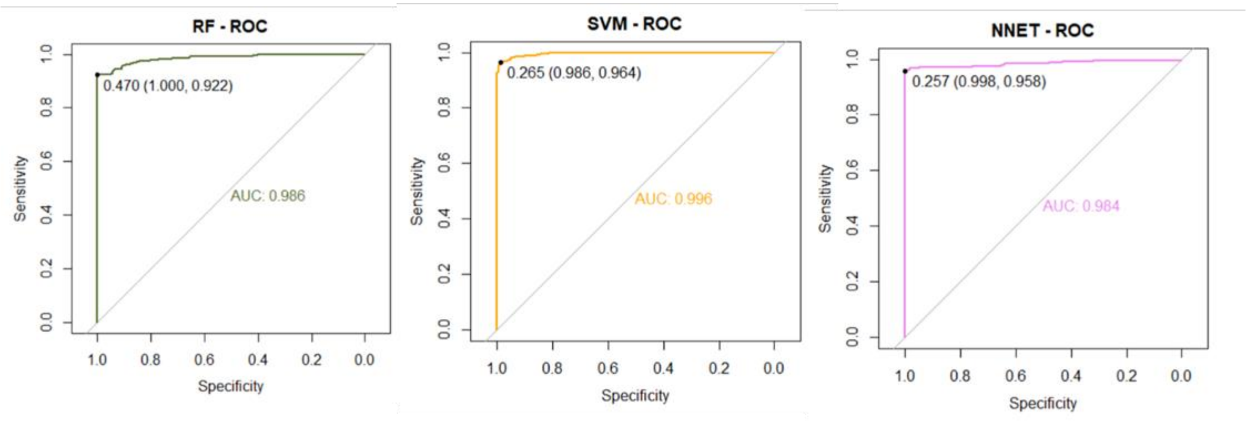

Supplementary Figure 17 a-c. Ogliarola salentina (OX) vs Leccino (LX) in *Xf* infected samples. ROC (Receiving Operator Characteristics) curves of the trained model applied on training set. The turning slope point represent the best threshold, with the optimal couple of Specificity and Sensitivity as coordinate. AUC is the Area Under Curve.

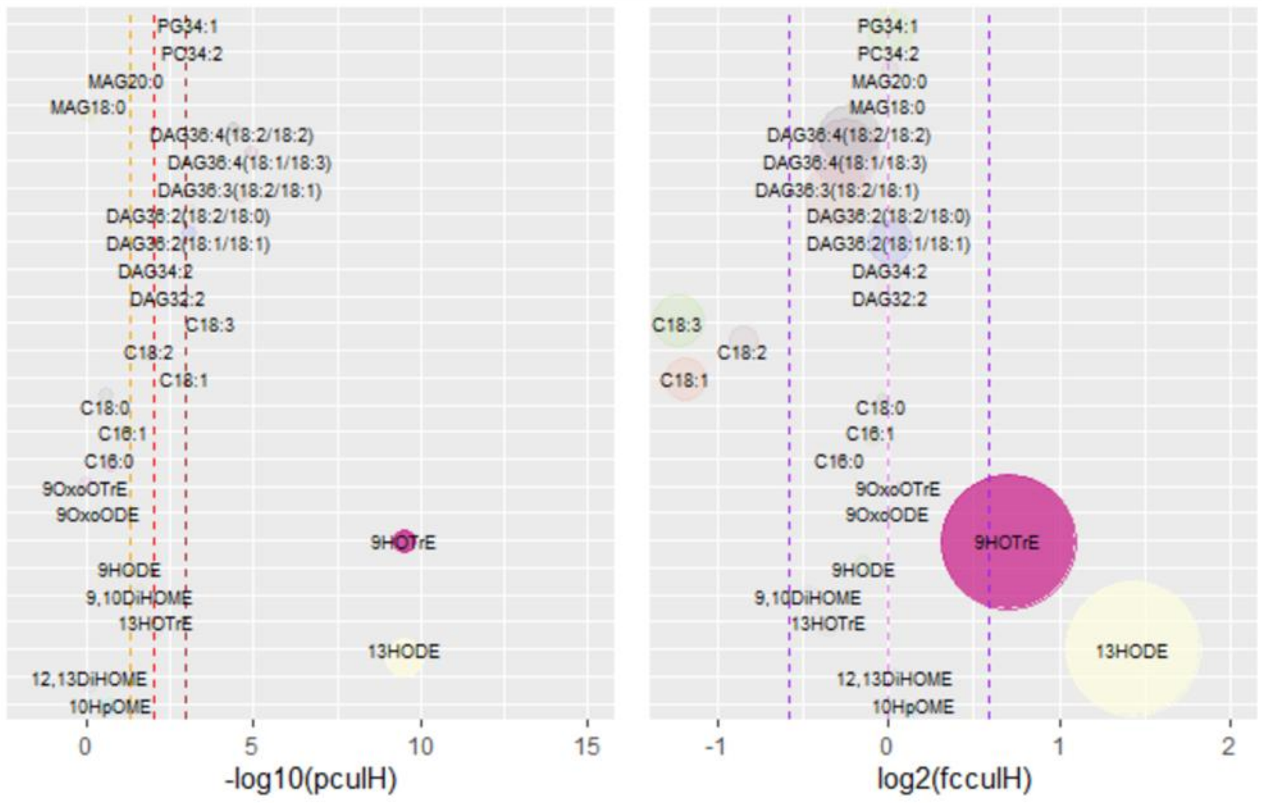

Supplementary Figure 18. Ogliarola salentina (OH) vs Leccino (LH) in *Xf* negative samples. Univariate statistical analysis- Statistical significance (Wilcoxon Mann Whitney test) and fold change of lipid entities. The left panel shows statistically significant compounds: on x-axis there is the -log10(p-value), on y-axis compounds name. The vertical dashed lines correspond to, respectively, -log10(0.05), -log10(0.01), -log10(0.001). Compounds under the first threshold are represented as transparent, compounds above the threshold are in full colors. Circle dimensions are proportional to fold changes. On right panel x-axis there is instead the log2(fold-change), on y-axis again compounds name. The right panel point out compounds with the biggest fold changes, represented in full colors, while compounds with a fold change>=1.5 or <=1.5 are transparent. Circle dimension are proportional to -log10(p-value).

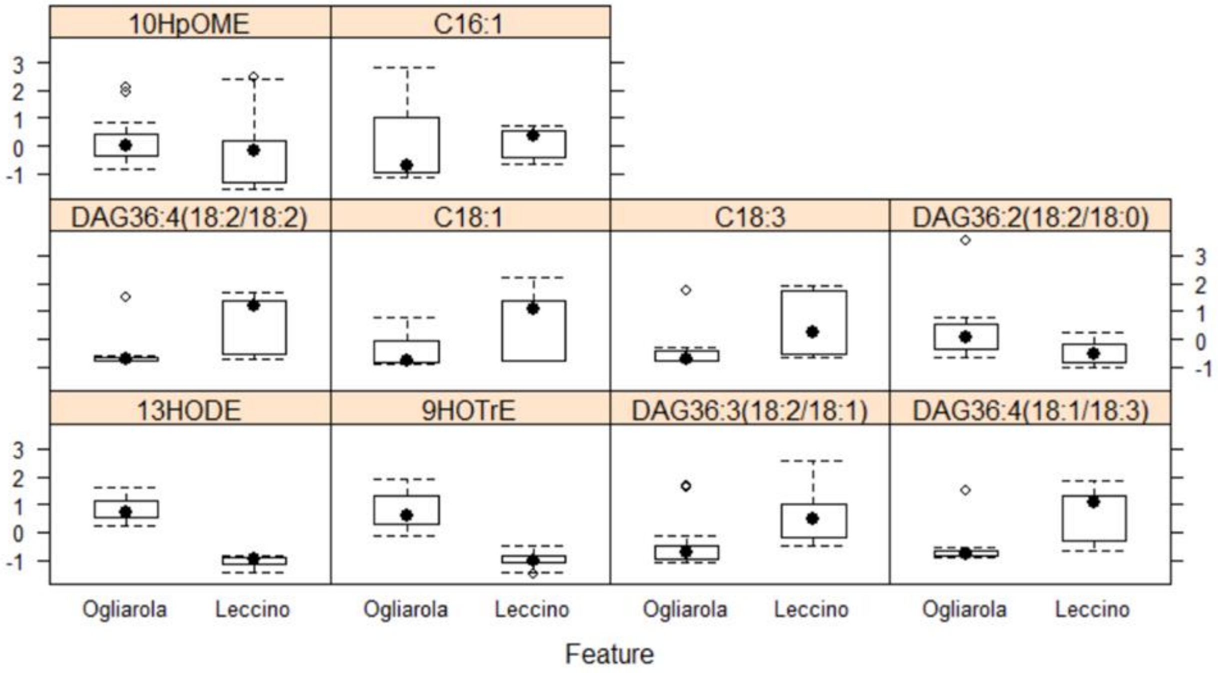

Supplementary Figure 19. Ogliarola salentina (OH) vs Leccino (LH) in *Xf* negative samples. Machine Learning Analysis. Feature selection: top 10 predictors found by Random Forest algorithm. Values on y-axis represent relatives amount of compounds. These values are peak areas normalize on IS and then centered and scaled before machine learning analysis

a) b) c)

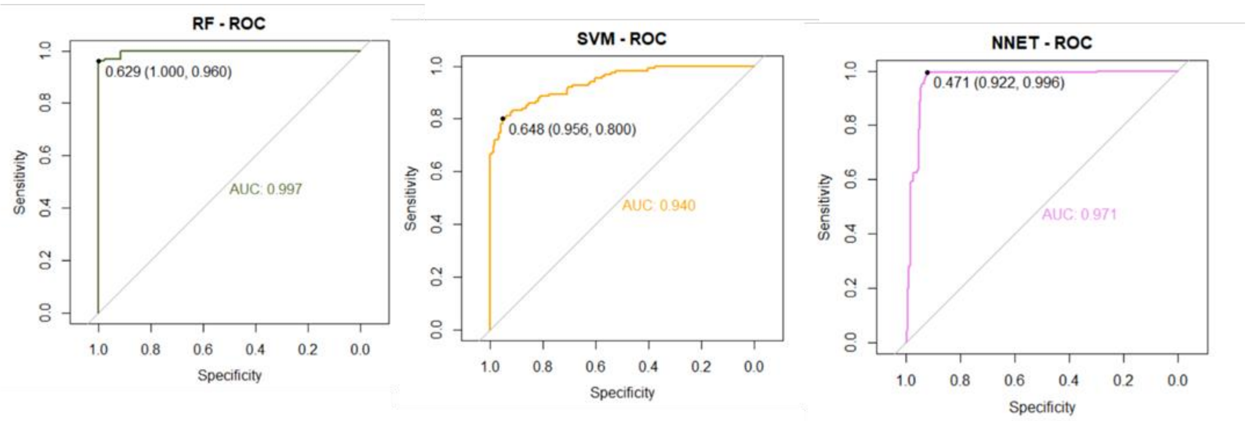

Supplementary Figure 20 a-c. Ogliarola salentina (OH) vs Leccino (LH) in *Xf* negative samples. ROC (Receiving Operator Characteristics) curves of the trained model applied on training set. The turning slope point represent the best threshold, with the optimal couple of Specificity and Sensitivity as coordinate. AUC is the Area Under Curve.

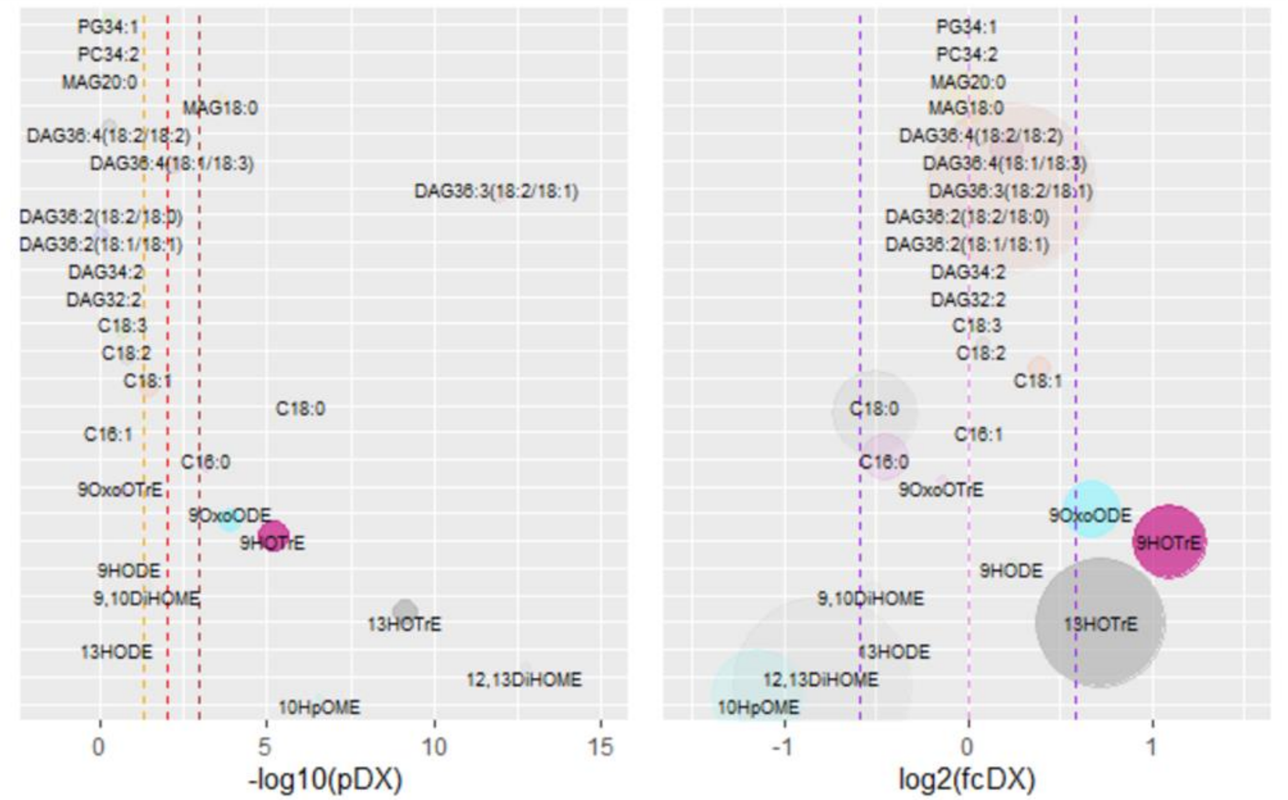

Supplementary Figure 21. Dentamet(R) treated (DX) vs untreated (NDX) in *Xf* positives samples. Univariate statistical analysis. Statistical significance (Wilcoxon Mann Whitney test) and fold change of lipid entities. The left panel shows statistically significant compounds: on x-axis there is the -log10(p-value), on y-axis compounds name. The vertical dashed lines correspond to, respectively, -log10(0.05), -log10(0.01), -log10(0.001). Compounds under the first threshold are represented as transparent, compounds above the threshold are in full colors. Circle dimensions are proportional to fold changes. On right panel x-axis there is instead the log2(fold-change), on y-axis again compounds name. The right panel point out compounds with the biggest fold changes, represented in full colors, while compounds with a fold change>=1.5 or <=1.5 are transparent. Circle dimension are proportional to -log10(p-value).

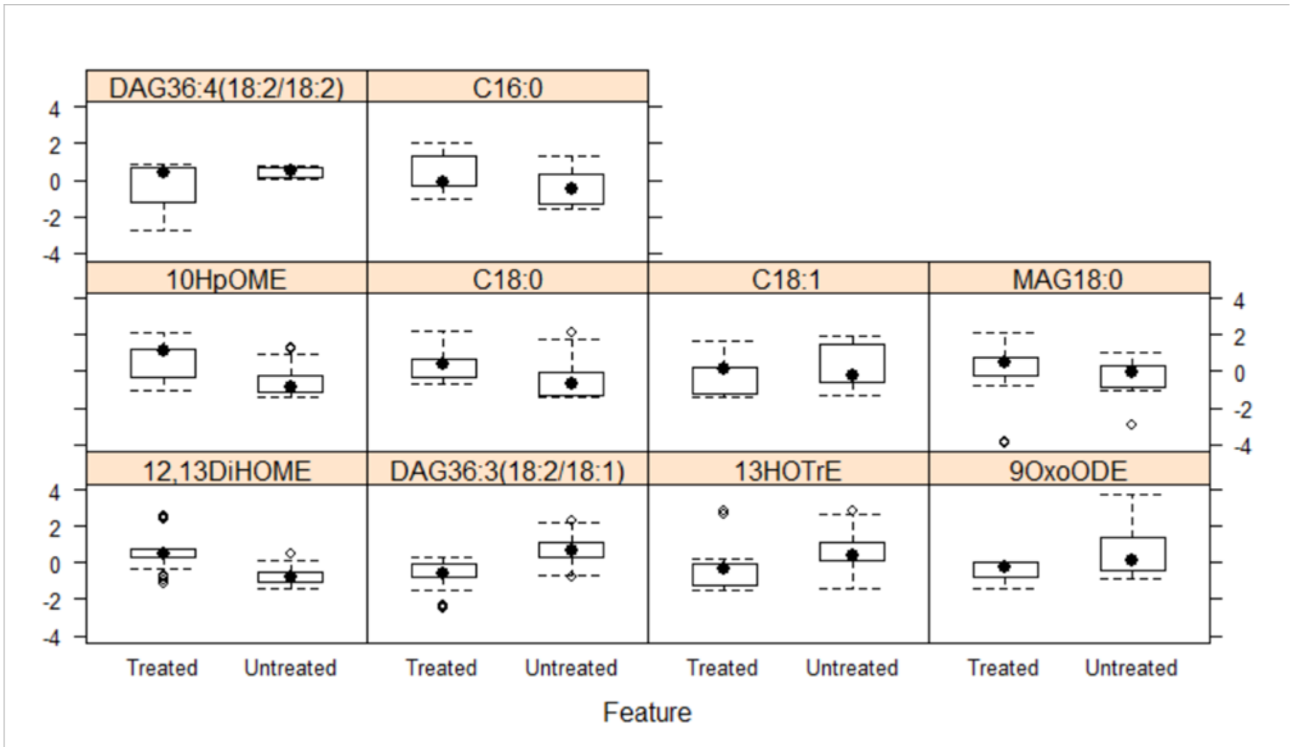

Supplementary Figure 22. Dentamet[(R)] treated (DX) vs untreated (NDX) in *Xf* positives samples. Machine Learning Analysis.Feature selection: top 10 predictors found by Random Forest algorithm. Values on y-axis represent relatives amount of compounds. These values are peak areas normalize on IS and then centered and scaled before machine learning analysis

a) b) c)

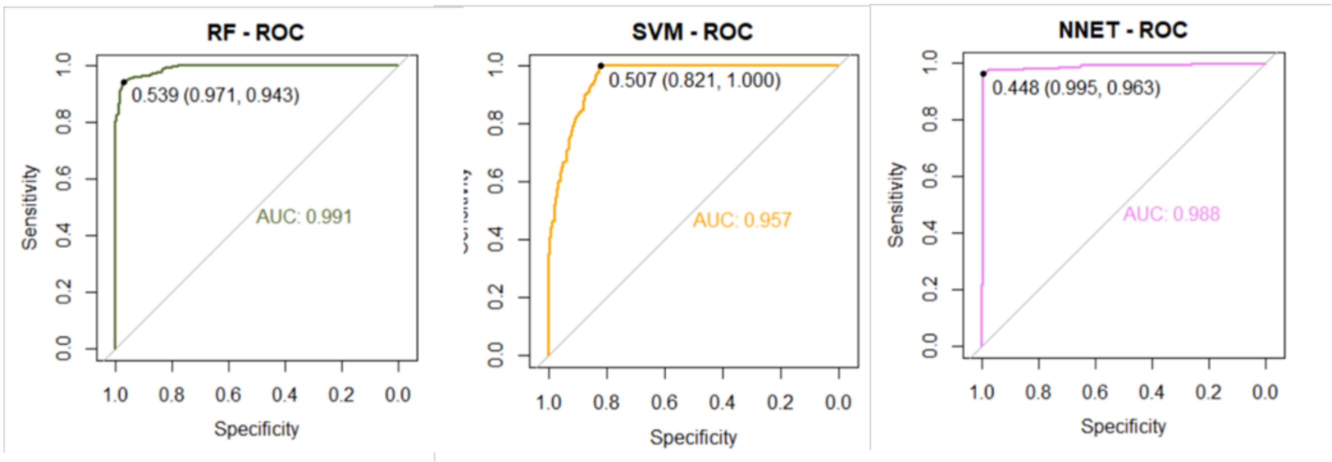

Supplementary Figure 23 a-c. Dentamet(R) treated (DX) vs untreated (NDX) in *Xf* positives samples. ROC (Receiving Operator Characteristics) curves of the trained model applied on training set. The turning slope point represent the best threshold, with the optimal couple of Specificity and Sensitivity as coordinate. AUC is the Area Under Curve

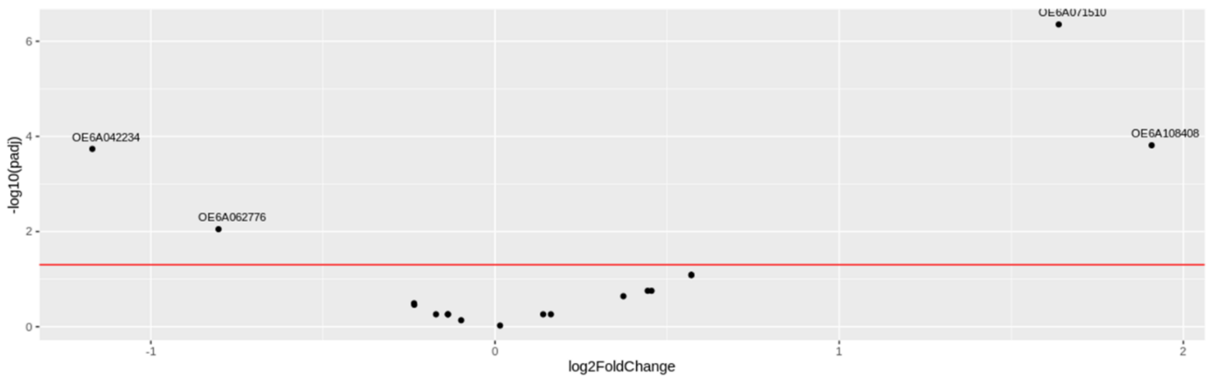

Supplementary Figure 24. Volcano plot of OXvsLX comparison. X-axis: log2 of gene fold change. Y-axis: -log10 of adjusted p-value. Vertical red dashed line is set on x=0, separating down regulated genes (left side) from up regulated (right side). Horizontal red dashed line is set on y=-log100.05, as a threshold for statistically significant genes.

1.2 Supplementary Tables

Supplementary Table 1. Summary of metrics used to assess machine learning models on test sets.

Comparison	Model	Sensitivity	Specificity	Precision	Balanced Accuracy
XvsH	RF	0.857143	0.921053	0.800000	0.889098
XvsH	SVM	0.642857	0.921053	0.750000	0.781955
XvsH	NNET	0.714286	0.921053	0.769231	0.817669
OXvsOH	RF	1.000000	0.888889	0.800000	0.944444
OXvsOH	SVM	1.000000	0.888889	0.800000	0.944444
OXvsOH	NNET	1.000000	0.888889	0.800000	0.944444
LXvsLH	RF	0.666667	0.800000	0.500000	0.733333
LXvsLH	SVM	0.666667	0.800000	0.500000	0.733333
LXvsLH	NNET	0.666667	0.800000	0.500000	0.733333
OvsL	RF	0.961538	0.807692	0.833333	0.884615
OvsL	SVM	0.884615	1.000000	1.000000	0.942308
OvsL	NNET	0.961538	1.000000	1.000000	0.980769
OXvsLX	RF	0.777778	0.850000	0.823529	0.813889
OXvsLX	SVM	0.777778	0.800000	0.777778	0.788889
OXvsLX	NNET	0.777778	0.900000	0.875000	0.838889
OHvsLH	RF	1.000000	0.666667	0.800000	0.833333
OHvsLH	SVM	1.000000	1.000000	1.000000	1.000000
OHvsLH	NNET	1.000000	1.000000	1.000000	1.000000
DXvsDNX	RF	1.000000	1.000000	1.000000	1.000000
DXvsDNX	SVM	0.700000	1.000000	1.000000	0.850000
DXvsDNX	NNET	1.000000	1.000000	1.000000	1.000000

Supplementary Table 2. *Xf*-infected (X) versus Healthy (H) samples. Predictors selected for Machine learning analysis. Importance evaluated through AUC.

**Important Features**	**AUC**
DAG36:4(18:1/18:3)	0.966301
DAG36:4(18:2/18:2)	0.965517
C18:2	0.956113
DAG36:3(18:2/18:1)	0.934169
C18:3	0.934169
C18:1	0.922414
13-HOTrE	0.880094
C16:1	0.863636
9-HODE	0.851881
13-HODE	0.846395

Supplementary Table 3. *Xf*-infected (OX) versus Healthy (OH) samples in Ogliarola salentina. Predictors selected for Machine learning analysis. Importance evaluated through AUC.

**Important Features**	**AUC**
13-HOTrE	1
13-HODE	1
DAG36:4(18:1/18:3)	1
C18:3	0.994048
C18:1	0.994048
C18:2	0.994048
DAG36:4(18:2/18:2)	0.994048
DAG36:3(18:2/18:1)	0.991071
9-HODE	0.979167
9-OxoODE	0.931548

Supplementary Table 4. *Xf*-infected (LX) versus Healthy (LH) samples in Leccino. Predictors selected for Machine learning analysis. Importance evaluated through AUC.

**Important Features**	**AUC**
DAG36:3(18:2/18:1)	1
DAG36:4(18:1/18:3)	1
DAG36:4(18:2/18:2)	0.996667
C18:2	0.993333
C18:3	0.933333
13-HODE	0.926667
C18:1	0.88
C16:1	0.866667
9-OxoOTrE	0.786667
9-HOTrE	0.776667

Supplementary Table 5. Ogliarola salentina (O) vs Leccino (L)**.** Predictors selected for Machine learning analysis. Importance evaluated through AUC.

**Important Features**	**AUC**
13-HODE	0.92125
9-HOTrE	0.659375
DAG36:3(18:2/18:1)	0.55875
DAG36:4(18:2/18:2)	0.54875
C18:2	0.547188
DAG36:4(18:1/18:3)	0.546875
C18:3	0.540313
C18:1	0.539063
9-OxoODE	0.519688
9-HODE	0.505938

Supplementary Table 6. Ogliarola salentina (OX) vs Leccino (LX) in *Xf* infected samples. Predictors selected for Machine learning analysis. Importance evaluated through AUC.

Important Features	AUC
13-HODE	0.982143
C18:0	0.729762
12,13-DiHOME	0.679762
DAG36:3(18:2/18:1)	0.669048
9-HODE	0.658333
DAG36:4(18:1/18:3)	0.619048
9-OxoODE	0.597619
DAG36:4(18:2/18:2)	0.595238
C18:1	0.583333
10-HpOME	0.533333

Supplementary Table 7. Ogliarola salentina (OH) vs Leccino (LH) in *Xf* negative samples. Predictors selected for Machine learning analysis. Importance evaluated through AUC.

Important Features	AUC
13-HODE	1
9-HOTrE	1
DAG36:4(18:1/18:3)	0.916667
C18:1	0.916667
DAG36:3(18:2/18:1)	0.908333
DAG36:4(18:2/18:2)	0.908333
C18:3	0.883333
DAG36:2(18:2/18:0)	0.725
C16:1	0.658333
10-HpOME	0.525

Supplementary Table 8. Dentamet(R) treated (DX) vs untreated (NDX) in *Xf* positives samples. Predictors selected for Machine learning analysis. Importance evaluated through AUC.

Important Features	AUC
12,13-DiHOME	0.914352
DAG36:3(18:2/18:1)	0.912616
13-HOTrE	0.810185
10-HpOME	0.769676
9-OxoODE	0.768519
C18:0	0.744213
C18:1	0.607639
DAG36:4(18:2/18:2)	0.625000
13-HODE	0.616898
C16:0	0.726852

Supplementary Table 9. Hypothetical Lipoxygenases found differentially expressed in OXvsLX.

	Gene ID	ProtEC	ProtDefinition	baseMean	log2FoldChange	lfcSE	pvalue	padj
1	OE6A005786	EC:1.13.11	linoleate 13S-lipoxygenase 2-1, chloroplastic-like	0	NA	NA	NA	NA
2	OE6A009273	EC:1.13.11	probable linoleate 9S-lipoxygenase 5 isoform X2	885,253977518553	0,454734991917408	0,312558232729864	0,0725947442696919	0,1763015217978223
5	OE6A027286	EC:1.13.11.12	linoleate 13S-lipoxygenase 3-1, chloroplastic	3576,74697604176	-0,137092149374108	0,183071233296052	0,427794411968718	0,5500877508803 81
6	OE6A030381	EC:1.13.11	linoleate 9S-lipoxygenase 6-like	0	NA	NA	NA	NA
7	OE6A031033	EC:1.13.11	probable linoleate 9S-lipoxygenase 5 isoform X2	164,945038265513	0,014619871057843	0,278102638816005	0,945901214094242	0,9459012140942 42
10	OE6A033601	EC:1.13.11	probable linoleate 9S-lipoxygenase 5 isoform X2	138,653505951313	-0,136840446049178	0,217842830561483	0,485371544894454	0,5500877508803 81
11	OE6A034518	EC:1.13.11	lipoxygenase 6, chloroplastic	841,134232525982	0,1622687705341 8	0,237014294971564	0,447962331590094	0,5500877508803 81
12	OE6A042234	EC:1.13.11	probable linoleate 9S-lipoxygenase 5	1212,3037652131	-1,1701724227457 4	0,317550047030911	3,2391658959414 4e-05	0,000183552734103349

13	OE6A043608	EC:1.13.11.12	linoleate 13S-lipoxygenase 3-1, chloroplastic	10712,342951202	-0,234636661328691	0,20088630119012	0,204222860328176	0,347178862557899
14	OE6A049116	EC:1.13.11	probable linoleate 9S-lipoxygenase 5 isoform X2	272,227561059763	0,372955189275653	0,271078004129685	0,107779301661808	0,229031016031342
16	OE6A050816	EC:1.13.11.12	linoleate 13S-lipoxygenase 3-1, chloroplastic	4684,90701912909	-0,235248199297489	0,184189629153839	0,170680430733287	0,322396369162876
17	OE6A062776	EC:1.13.11	probable linoleate 9S-lipoxygenase 5	13167,8708517889	-0,80347774355979	0,309560369825094	0,00209880942601501	0,00891994006056379
18	OE6A071510	EC:1.13.11	linoleate 13S-lipoxygenase 2-1, chloroplastic-like	198,001717991757	1,6379030921176	0,31743385299594	2,60283596795071e-08	4,42482114551621e-07
20	OE6A072146	EC:1.13.11	linoleate 9S-lipoxygenase 6-like	39,3554682816546	-0,0984709867176662	0,323868952187786	0,689281066899846	0,732361133581086
21	OE6A073273	EC:1.13.11	probable linoleate 9S-lipoxygenase 5 isoform X2	856,886456032096	0,443572291842406	0,291931517463683	0,0701338559737991	0,176301521797823
22	OE6A079300	EC:1.13.11	linoleate 9S-lipoxygenase 6-like	0,587460273885676	0,13981686843484	0,539193985230092	0,418088306440079	0,550087750880381
23	OE6A080778	EC:1.13.11	probable linoleate 9S-lipoxygenase 5 isoform X2	783,088670274443	0,570387096461182	0,30490629377978	0,0240674972295605	0,0818294905805056
24	OE6A080778	EC:1.13.11	probable linoleate 9S-lipoxygenase 5 isoform X1	783,088670274443	0,570387096461182	0,30490629377978	0,0240674972295605	0,0818294905805056

25	OE6A091158	EC:1.13.11.12	lipoxygenase 6, chloroplastic	4,121314807868 91	-0,1714935860368 64	0,474686463011593	0,46451772916843 7	0,550087750880381
27	OE6A108408	EC:1.13.11	linoleate 13S-lipoxygenase 2-1, chloroplastic-like	14,1262802641262	1,90786431932845	0,515154586755232	1,80833126109278e-05	0,000153708157192886
